# Genomic Data Reveals Population Genetic and Demographic History of *Magnolia fistulosa* (Magnoliaceae), a Plant Species With Extremely Small Populations in Yunnan Province, China

**DOI:** 10.3389/fpls.2022.811312

**Published:** 2022-02-17

**Authors:** Fengmao Yang, Lei Cai, Zhiling Dao, Weibang Sun

**Affiliations:** ^1^Yunnan Key Laboratory for Integrative Conservation of Plant Species With Extremely Small Populations, Kunming Institute of Botany, Chinese Academy of Sciences, Kunming, China; ^2^Key Laboratory for Plant Diversity and Biogeography of East Asia, Kunming Institute of Botany, Chinese Academy of Sciences, Kunming, China; ^3^University of the Chinese Academy of Sciences, Beijing, China

**Keywords:** *Magnolia fistulosa*, ddRAD-seq, population genetic, demographic history, conservation management

## Abstract

Elucidating the genetic background of threatened species is fundamental to their management and conservation, and investigating the demographic history of these species is helpful in the determination of the threats facing them. The woody species of the genus *Magnolia* (Magnoliaceae) have high economic, scientific and ecological values. Although nearly half of all *Magnolia* species have been evaluated as threatened, to date there has been no population genetic study employing Next Generation Sequencing (NGS) technology in this genus. In the present study, we investigate the conservation genomics of *Magnolia fistulosa*, a threatened species endemic to the limestone area along the Sino-Vietnamese border, using a double digest restriction-site-associated DNA-sequencing (ddRAD-seq) approach. To increase the reliability of our statistical inferences, we employed two approaches, Stacks and ipyrad, for SNP calling. A total of 15,272 and 18,960, respectively, putatively neutral SNPs were generated by Stacks and ipyrad. Relatively high genetic diversity and large population divergence were detected in *M. fistulosa*. Although higher absolute values were calculated using the ipyrad data set, the two data sets showed the same trends in genetic diversity (π, *H*_e_), population differentiation (*F*_ST_) and inbreeding coefficients (*F*_IS_). A change in the effective population size of *M. fistulosa* within the last 1 Ma was detected, including a population decline about 0.5–0.8 Ma ago, a bottleneck event about 0.2–0.3 Ma ago, population fluctuations during the last glacial stage, and the recovery of effective population size after the last glacial maximum. Our findings not only lay the foundation for the future conservation of this species, but also provide new insights into the evolutionary history of the genus *Magnolia* in southeastern Yunnan, China.

## Introduction

Endangered species, especially those with extremely small populations, often face a high risk of extinction under climatic fluctuations and anthropogenic disturbance (Bijlsma and Loeschcke, 2012; Ralls et al., 2018). In addition to reproduction difficulties as a result of low number of individuals (Kéry et al., 2000), small populations can be subject to genetic drift, which can erode genetic diversity and lead to rapidly increasing genetic divergence between populations in the long term (Willi et al., 2007; Haag et al., 2010). Loss of genetic diversity may reduce population persistence and the evolutionary potential of species, and high genetic differentiation may lead to outbreeding depression (Elam, 1993; Spielman et al., 2004; Bomblies and Weigel, 2007).

Plant Species with Extremely Small Populations (PSESP) is a conservation concept referring to plant species with small remaining populations (<5,000 individuals), restricted habitat, exposure to serious human disturbance, and at high risk of extinction (Ma et al., 2013; Sun et al., 2019a; Yang et al., 2020). Indeed, PSESP are the most critically endangered of all species and urgently need protection (Cai et al., 2019; Crane, 2020; Li et al., 2020). The protection and maintenance (both *in situ* and *ex situ*) of different evolutionarily significant units identified in threatened species will maximize their evolutionary potential to handle environmental change (Funk et al., 2012). Therefore, investigation of the genetic background and population structure of PSESPs is fundamental to their conservation and management (Liu et al., 2020; Yang et al., 2020; Ma et al., 2021a,b).

Advances in sequencing technology, especially reduced-representation genome sequencing, have allowed us to obtain thousands of genetic markers relatively easily (Allendorf et al., 2010), making it possible to answer many evolutionary questions and guide conservation (Andrews et al., 2016; Beichman et al., 2018). Restriction-Site Associated DNA sequencing (RAD-seq) technology is one of the most promising technologies in conservation genetics, as it is capable of producing an abundance of genetic markers with or without a reference genome in an economical way (Andrews et al., 2016). RAD-seq has a huge advantage over simple sequence repeats (SSR) data, in that the resolution of data sets is increased, no prior genetic information is needed and the inference of demographic history provided is thought to be more accurate (Jeffries et al., 2016; Lemopoulos et al., 2019). Compared with traditional RAD-seq, double digest RAD sequencing (ddRAD-seq) further improves efficiency and robustness while minimizing cost (Peterson et al., 2012). However, several studies have shown that the choice of pipeline used for the analysis may have a significant impact on the accuracy of the results (Qi et al., 2015; Torkamaneh et al., 2016; Boukteb et al., 2021). To improve the reliability of the results, multi-method analyses can be adopted to process data using different technical routes (Qi et al., 2015; Boukteb et al., 2021).

The genus *Magnolia* belongs to the family Magnoliaceae and is well known for its basal position in the flowering plants (The Angiosperm Phylogeny Group et al., 2016). The genus is mainly distributed throughout the temperate and tropical regions of Southeast Asia, the Antilles and Central and South America, and more than 300 species have been described to date (Law et al., 1995; Azuma et al., 2001; Law, 2004; Cires et al., 2013). China is the center of diversity for *Magnolia*, and is home to more than 160 *Magnolia* species, including the largest number of threatened *Magnolia* species of any country in the world (Law, 2004; Cires et al., 2013). *Magnolia* species are valued for their timber, culinary use, and their ornamental and medicinal applications (Cires et al., 2013), and overexploitation for these purposes has contributed to declines in the populations of many *Magnolia* species. Furthermore, strong human disturbance poses a huge threat to the survival of *Magnolia*. On the global scale, half of the *Magnolia* species are considered threatened, nevertheless, the conservation status of nearly a third of all *Magnolia* species remains unassessed (Rivers et al., 2016). Of the 160 *Magnolia* species found in China, 76 are considered to be threatened in the Threatened Species List of China’s Higher Plants (Qin et al., 2017). Because of the urgent conservation action required for many *Magnolia* species, there is much research focusing on the population genetic and demographic histories of threatened *Magnolia* (Isagi et al., 2007; Cires et al., 2013; Budd et al., 2015; Tamaki et al., 2019; Hernández et al., 2020). However, to date we know of no studies adopting a RAD-seq approach to study conservation genetics in *Magnolia*.

**FIGURE 1:**
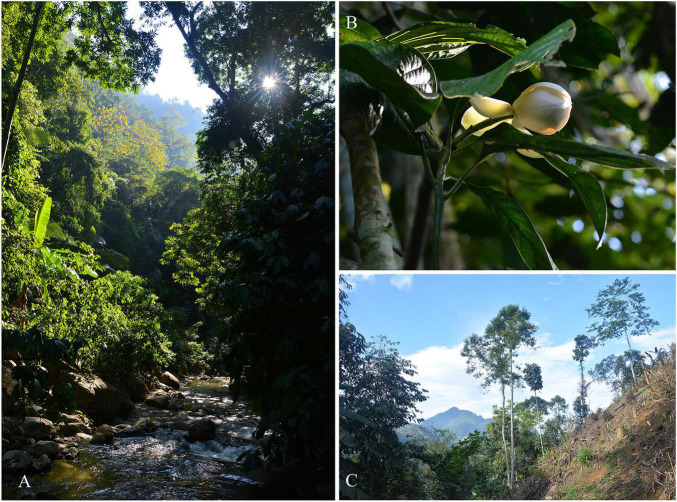
**(A)** The habitant of *Magnolia fistulosa* in QSH (Qingshui River, Gejiu). **(B)** The flower of *M. fistulosa*. **(C)** Habitat destruction of *M. fistulosa* in LB (Longbao Village, Hekou).

*Magnolia fistulosa* (Finet & Gagnepain) Dandy is an evergreen shrub with broad oval leaves and extremely aromatic flowers (Figure 1B). It is a threatened species and is endemic to the Sino-Vietnamese border (Xia, 2006; Deng, 2019). *M. fistulosa* was evaluated as Vulnerable in the Threatened Species List of China’s Higher Plants (Qin et al., 2017), however, in the Red List of Magnoliaceae (Rivers et al., 2016), it was evaluated as Data Deficient. *M. fistulosa* was also classified as a provincial PSESP in the Planning Outline of Rescuing and Conserving Yunnan’s PSESP (2010–2020) in 2010 by The Yunnan Provincial Government (Sun, 2013; Sun et al., 2019b). *M. fistulosa* is a tropical rain-forest plant (Figure 1A), and these forests also provide a refuge for other PSESPs, including *M. lucida*, *Hopea chinensis* (Dipterocarpaceae) and *Cycas dolichophylla* (Cycadaceae; Zheng et al., 2016; Sun et al., 2019b). During the course of our field investigations, we found a total of 245 mature *M. fistulosa* individuals in southeastern Yunnan. The pollinator of *Magnolia fistulosa* is *Fruhstorferia anthracina* (Rutelidae), a large (4 cm long) species of beetle. The main distribution of *M. fistulosa* was found to be in Hekou and Maguan Counties, while small numbers of disjunct individuals were also found in Gejiu and Jinping Counties. Throughout the range of *M. fistulosa*, there are varying degrees of human disturbance, such as crop planting and road construction (Figure 1C), with the threats facing the Jinping location being the worst. In Jinping County, only four individuals were found in remaining patches of forest and surrounded by rubber plantations. To date, there has been no research into the conservation genetics of *M. fistulosa*. Investigation of the population structure and genetic diversity of *M. fistulosa* would illustrate which factors might have shaped its PSESP status. Furthermore, the study would provide an example for the study of the demographic history of plants from southeastern Yunnan, and would, at the same time, lay the foundations of the conservation and management of *M. fistulosa*.

In this study, we use two different pipelines to process the sequence data generated from the double digested restriction site-associated DNA sequencing (ddRAD-seq) from *Magnolia fistulosa* taken from seven sampling locations. The genome of *Magnolia sinica* (BioProject ID PRJNA774088) was adopted as reference genome to improve the quantity and quality of the SNPs makers. In this study, we reveal the genetic threats currently faced by *M. fistulosa* and lay the foundation for the future conservation and management of this species. We aim to (1) explore the population structure of *M. fistulosa* and infer the levels and direction of gene flow among populations; (2) estimate the genetic diversity and contemporary effective population size of each population; and (3) infer the demographic history of *M. fistulosa* and its potential association with climatic fluctuations.

## Materials and Methods

### Sampling, DNA Extraction and Sequencing

**FIGURE 2:**
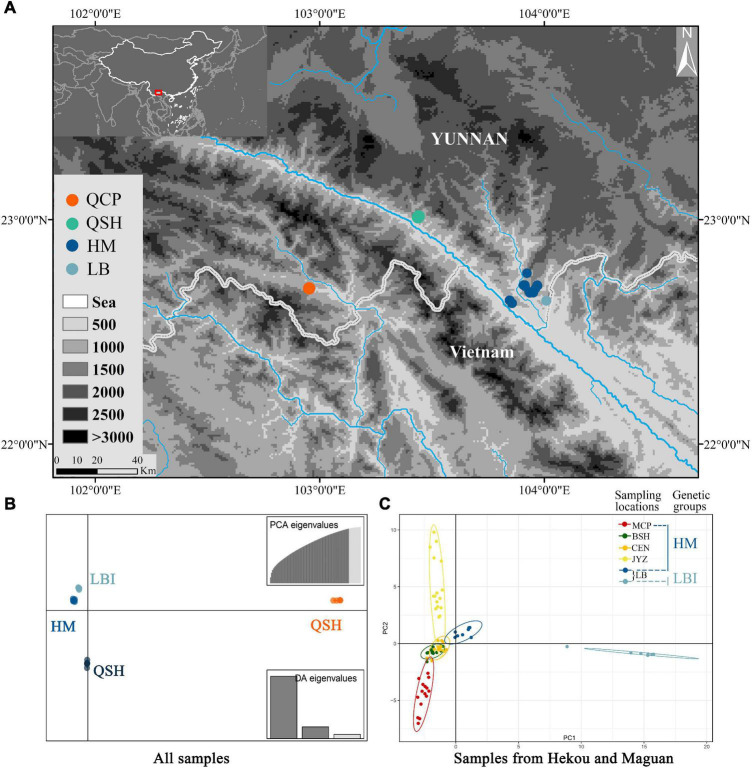
**(A)** Map of all sampling locations; HM represents the sampling locations of MCP, BSH, CEN and JYZ. **(B)** DAPC results from the Stacks data set with all populations and **(C)** PCA results from the Stacks data set with samples from Hekou and Maguan: HM and LBI are two genetic groups divided by DAPC analysis using samples from Hekou and Maguan.

We sampled a total of 93 *Magnolia fistulosa* individuals from seven sampling locations (MCP, BSH, CEN, JYZ, LB, QSH, and QCP; Figure 2A), and, with the exception of Jinping County (four individuals), 12–16 individuals were sampled from each sampling location. The distance between each sample is at least 20 m. Sampling locations of *M. fistulosa* covered every location in China where this species is known to occur.

Total DNA was extracted from silica gel-dried leaf tissues using a CTAB method (Doyle and Doyle, 1990). The quantity and quality of each sample was tested using agarose gel (Omega Bio-Tek, Norcross, GA, United States) electrophoresis and on a fluorometer (qubit3.0, Thermo Fisher Scientific, Waltham, MA, United States). Each qualified DNA sample was standardized to the same volume (10 μl) and quantity of DNA (200 μg). A 10 μl pre-mixed double enzyme (EcoRI and MseI; New England Biolabs, Ipswich, MA, United States) digestion solution was added to each sample. Samples were then placed in a PCR machine (Takara PCR Thermal Cycler Dice, Takara Bio Inc., Shiga, Japan) for 8 h at 37°C, followed by 20 min at 65°C, with a final incubation step at 12°C. The digested fragments were detected using agarose gel electrophoresis. Digested fragments were ligated to EcoRI and MseI adapters containing sample specific barcodes using a PCR machine with the following conditions: 16°C for 8 h, 65°C for 20 min, and 12°C for final incubation. Barcoded samples were separated by size (350–500 bp) using an agarose gel. To meet the concentration requirements for sequencing, each library was then amplified using a PCR machine with the following conditions: 98°C for 2 min, followed by 25 cycles for 98°C for 15 s, 56°C for 15 s and 72°C for 15 s; and 72°C for 5 min. The paired-end sequencing was performed on an Illumina Hiseq X-Ten platform (Illumina Inc., San Diego, CA, United States).

### Processing of Next Generation Sequencing Data

Reference genomes were used to improve the accuracy of SNP calling. Three assembled genomes at chromosome-level, *Magnolia sinica* (BioProject ID PRJNA774088), *M. officinalis* (Yin et al., 2021) and *M. biondii* (Dong et al., 2021) were compared. To process reduced-representation genome sequencing data into SNP genotypes, two pipelines are commonly used. We adopted both of these to cross-validate our results from the population genetic statistics and population structure analyses.

The Stacks v2.55 pipeline (Catchen et al., 2013; Rochette and Catchen, 2017) was employed to process the reads generated by ddRAD-seq. The raw data was filtered and demultiplexed using process_radtags with the len_limit set to 140 bp to trim low-quality reads, and retain_header -t was set to 135. We used BWA v07.17 (Li and Durbin, 2009) to make an index for the three reference genomes and used BWA-MEM with the default parameters to map the filtered reads to the three reference genomes separately. The mapping rates for each sample to each of the three genomes were calculated using -flagstat in SAMtools v1.7 (Li et al., 2009; Danecek et al., 2021), and the genome with highest mapping rates was chosen to process the later steps. We used gstacks to identify SNPs within the meta population at each locus, and then to genotype each sample at each identified SNP. The populations module in the Stacks pipeline was used to call and filter the SNPs. To reduce the missing rate of SNP matrix, we used the filtering parameter −*r* = 0.8, in which not less than 80% of individuals in a population were required to possess a locus. The threshold parameters –min-maf = 0.01 was set to improve the accuracy of the model based population structure analyses (Linck and Battey, 2019). To exclude closely linked loci and to keep only the first SNP per ddRAD-seq locus, we used -write-single-snp. Finally, we only keep the SNPs mapped to the chromosomes.

We also used the ipyrad v.0.9.81 pipeline (Eaton and Overcast, 2020) to process the SNP calling task. Filtered by process_radtags in the Stacks pipeline, clean reads were then processed with the following parameters in ipyrad: min depth for statistical base calling = 6, minimum taxon coverage = 75, max heterozygous sites per locus = 0.3. Using the same filtering criteria as in the Stacks pipeline, the SNPs were then filtered using VCFtools v0.1.16 (Danecek et al., 2011) using the following criteria: max missing rate > 0.8, minor allele frequency (MAF) > 0.01, only one SNP per locus kept.

Tajima’s D (Tajima, 1989) was calculated in VCFtools with 20,000 bp window sizes and 95% confidence limit to test all the loci for neutrality. We also detected selected SNP loci using “*F*_ST_ outlier” tests in BayeScan v2.1 (Foll and Gaggiotti, 2008), with a *q*-value lower than 1%. After filtering the SNP loci potentially under selection, putatively neutral sites were adopted to process the following analyses. We used VCFtools to find cross-validated SNPs shared by the two putatively neutral SNP data sets. Research suggests that SNPs called by more than one pipeline are typically accurate (Torkamaneh et al., 2016). PGDSpider v 2.1.1.5 (Lischer and Excoffier, 2011) was used for format conversion.

### Population Structure and Genetic Diversity

Population structure was inferred using Discriminant Analysis of Principal Components (DAPC), Principal Components Analysis (PCA) and Bayesian clustering. DAPC analysis was conducted using the R package “adegenet” (Jombart and Ahmed, 2011). The function find.clusters() in “adegenet” was used to find the *de novo* population structure and the optimal number of PCs. PCA analysis was also conducted using “adegenet,” both for all samples and for samples in Hekou and Maguan. Bayesian clustering was performed using Admixture (Alexander et al., 2009). To determine the optimal value of *K*, we tested numbers of clusters from two to seven, with the optimal number of clusters estimated via the lowest cross-validation error rate. We used the package “Pophelper” (Francis, 2017) in R v3.6.3 (R Core Team, 2018) to visualize the Admixture results. Recent gene flow among sampling locations was evaluated using the Bayesian inference approach BayesAss (Wilson and Rannala, 2003) as implemented in the BA3SNP program (Mussmann et al., 2019), with 1.0 × 10^7^ iterations, a burn-in of 1.0 × 10^6^ steps and a sampling frequency of 1,000. The cross-validated SNP data sets were used to improve the accuracy of evaluation. Adjustments were made in the two parameters -a and -f to make the acceptance rates for allele frequencies and inbreeding coefficients between 20 and 60% (Mussmann et al., 2019). AMOVA analysis was carried out in Arlequin v3.5 (Excoffier and Lischer, 2010) to quantify the genetic difference among and within the genetic groups inferred by the population structure analyses. To test for the presence of isolation-by-distance (IBD), we performed Mantel tests using the R package “ade4” (Dray and Dufour, 2007) with 10,000 permutations.

The nucleotide diversity (π), expected heterozygosity (*H*_e_), observed heterozygosity (*H*_o_), fixation index (*F*_ST_) and inbreeding coefficient (*F*_IS_) were calculated at each sampling location and genetic group using the populations module in the Stacks pipeline. Contemporary effective population size (*N*_e_) for each sampling location and each genetic group (sample size = n) was estimated using the linkage disequilibrium method in NeEstimator V2 (Do et al., 2014) with the minor allele frequency cutoff set within the following interval: 1/(2n) ≤ PCRIT ≤ 1/n (Waples and Do, 2010).

### Inference of Demographic History

We used Stairway plot 2 (Liu and Fu, 2020) to infer the demographic history of *Magnolia fistulosa* under a mutation rate of 4e-9 per locus per year (referring to *M. sinica*, unpublished data) and a generation time of 10 years, which was estimated from the cultivation records in Kunming Botanic Garden and Gulinqing Nature Reserve. We generated Site Frequency Spectra (SFS) for all samples and for each genetic group inferred from the population structure analyses. Controlling overfitting is important in the inference of demographic history (Liu and Fu, 2020), and we therefore generated both unfolded and folded SFS for *M. fistulosa* using the functions doSaf and realSFS in the ANGSD v 0.921 (Korneliussen et al., 2014) with the recommended filtering parameters -minMapQ 30 -minQ 20 and taking the genome of *M. sinica* as the ancestral state.

## Results

### Processing of Sequence Data

A total of 425 million reads were produced for all samples. After filtering out low quality reads and reads without RAD-tags, 412 million reads remained for processing (Supplementary Table 1). The sequencing depth of samples ranged from 4.2× (BP4) to 12.71× (BSH2), with a mean coverage of 7.49× (Supplementary Table 1). After filtering, each individual was mapped to three reference genomes. The average mapping rate to *Magnolia sinica* was 86.00%, which outperformed *M. officinalis* (84.77%) and *M. biondii* (84.93%) (Supplementary Table 2). *M. sinica* was therefore chosen for use in the analysis pipelines.

Using the reference-based analysis in the Stacks pipeline, we produced 15,274 SNPs (Stacks data set). The missing rates in the Stacks data set ranged from 0 to 30.8%, with a mean missing rate of 5.5% (Supplementary Table 3). Using the reference assembly method in the ipyrad pipeline, we produced 18,963 SNPs (ipyrad data set). The missing rate in the ipyrad data set ranged from 2.2 to 54.3%, with a mean missing rate of 14.2% (Supplementary Table 3). Compared to the Stacks pipeline, ipyrad generated more SNPs, however, the ipyrad data set had a higher average missing rate.

Tajima’s D statistic showed that no selected sites were present in either of the two data sets, while BayeScan detected two SNPs in each data set under positive selection and a single SNP under balancing selection in the ipyrad data set. After filtering, 15,272 and 18,960 putatively neutral SNPs were retained in the Stacks and ipyrad data sets, respectively (Supplementary Figure 1). After comparison, a total of 1,612 SNPs cross-validated by both data sets were found.

### Population Structure and Genetic Diversity

Discriminant Analysis of Principal Components analysis supported the presence of four genetic groups (Figure 2B and Supplementary Figure 2), with samples from QCP, QSH, and 7 of the individuals from LB each clustering in its own group (QCP, QSH, and LBI, respectively), and all the other individuals (sampling locations MCP, BSH, CEN, JYZ, and LBE, representing another 8 individuals from LB) forming one group (HM; Figure 2B). We also conducted DAPC analysis to find *de novo* clusters within the samples only from Hekou and Maguan, and found LBI was separated from HM (Supplementary Figures 3, 4). The conclusions of the PCA were congruent to those of DAPC, as they also indicated that LBI was strongly separated from other samples in Hekou and Maguan (HM; Figure 2C and Supplementary Figure 4).

**FIGURE 3:**
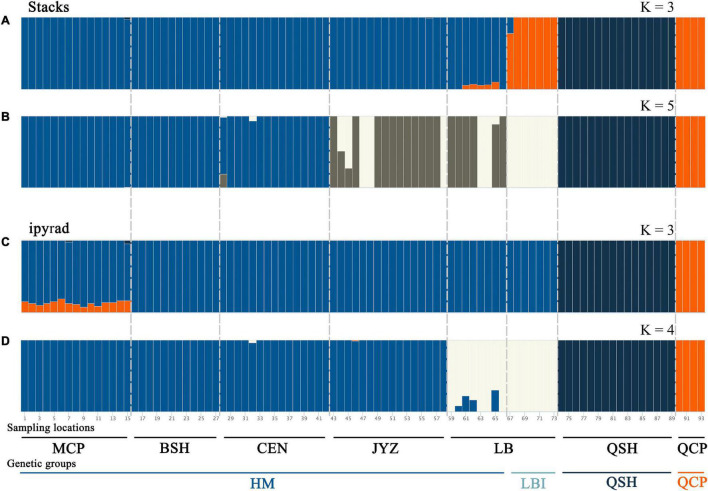
Population structure of *Magnolia fistulosa* inferred by two data sets. Admixture results with **(A)** *K* = 3 and **(B)** *K* = 5 in the Stacks data set, **(C)** *K* = 3 and **(D)** *K* = 4 in the ipyrad data set. The two lines represent each sampling location and the genetic cluster divided by DAPC and PCA analyses.

Bayesian clustering analysis was performed in Admixture. The two best two values of *K*, as indicated by CV error values, were 3 and 5 in the Stacks data set and 3 and 4 in the ipyrad data set (Supplementary Figure 5). Under *K* = 3, groups inferred from the Stacks data set and the ipyrad data set were congruent with the exception of LBI: whereas in the Stacks data set, LBI was joined with QCP in one genetic group (Figure 3A), in the ipyrad data set, it grouped with other samples from Hekou and Maguan (MCP, BSH, CEN, JYZ, and LBE; Figure 3C). In the *K* = 5 scenario from the Stacks data set (Figure 3B), the samples from Hekou and Maguan were further divided into three clusters, one including samples from MCP, BSH, and CEN, and the other two jointly including samples from LB and JYZ. In the *K* = 4 scenario from the ipyrad data set (Figure 3D) the samples from Hekou and Maguan were divided into two clusters, but in contrast to results from the Stacks data set JYZ was joined with MCP, BSH, and CEN in one cluster. Admixture analysis within the samples from Hekou and Maguan showed that the best number of clusters in both data sets was one (Supplementary Figures 5C,D). BayesAss analysis suggested that recent gene flow (over the last 1–3 generations) among sampling locations showed gene flow from JYZ to two genetic groups in LB, and gene flow from CEN to BSH and LBE (Table 1 and Supplementary Table 4). The Mantel test of geographical distance and genetic differentiation indicated that IBD is an important factor for the divergence of *Magnolia fistulosa* (*R*^2^ = 0.41, *P* < 1e-2 and *R*^2^ = 0.44, *P* < 1e-2 in two data sets; Supplementary Figure 6).

**TABLE 1:** Recent migration rates between each sampling location and the two genetic groups in LB using the cross-validated SNPs from the Stacks data set.

	Recipient of migration									

Source of migration	Sampling locations		MCP	BSH	CEN	JYZ	LB		QSH	QCP

							LBE	LBI		
	MCP		0.899	0.017	0.014	0.014	0.021	0.022	0.014	0.028
	BSH		0.014	0.841	0.015	0.014	0.021	0.022	0.014	0.028
	CEN		0.014	0.057	0.899	0.014	0.062	0.026	0.014	0.028
	JYZ		0.015	0.018	0.015	0.903	0.146	0.041	0.014	0.028
	LB	LBE	0.015	0.017	0.015	0.014	0.687	0.022	0.014	0.028
		LBI	0.014	0.017	0.014	0.014	0.021	0.822	0.014	0.028
	QSH		0.015	0.017	0.015	0.014	0.021	0.022	0.903	0.028
	QCP		0.014	0.017	0.015	0.014	0.021	0.022	0.014	0.806

The fixation index (*F*_ST_) showed that the genetic differences among samples in Hekou and Maguan were relatively small (*F*_ST_ < 0.15), while the genetic differences between QCP, QSH, and LBI were larger. QCP and LBI had the highest *F*_ST_ values, which were calculated to be 0.359 and 0.469 in the Stacks and ipyrad data sets, respectively (Table 2). AMOVA analysis revealed that 27.08 and 26.82% of the genetic variation was distributed between four genetic groups (HM, LBI, QSH, and QCP; Supplementary Table 5).

**TABLE 2:** Genetic differentiation coefficient (*F*_ST_) between each sampling location and the two genetic groups in LB.

Sampling locations		MCP	BSH	CEN	JYZ	LB		QSH	QCP

						LBE	LBI		
MCP			0.051	0.043	0.049	0.067	0.105	0.094	0.185
BSH		0.042		0.043	0.051	0.069	0.119	0.109	0.218
CEN		0.036	0.035		0.044	0.060	0.098	0.095	0.188
JYZ		0.041	0.043	0.037		0.063	0.101	0.095	0.188
LB	LBE	0.055	0.059	0.049	0.052		0.149	0.135	0.285
	LBI	0.086	0.097	0.079	0.082	0.123		0.204	0.469
QSH		0.069	0.079	0.069	0.069	0.098	0.148		0.295
QCP		0.127	0.151	0.127	0.129	0.205	0.359	0.195	

*The lower triangle is F_ST_ calculated for the Stacks data set, and the upper triangle is F_ST_ calculated for the ipyrad data set.*

The two data sets showed the same trends in all population genetic statistics, however, the ipyrad data set had higher absolute values than the Stacks data set (Table 3). From the Stacks data set, π ranged from 0.030 (QCP) to 0.067 (MCP), and was 0.072 at the species level. In the ipyrad data set, π ranged from 0.041 (QCP) to 0.084 (MCP) and was 0.097 at the species level. The HM genetic group exhibited the highest nucleotide diversity in both data sets (0.068 and 0.089 in the Stacks and ipyrad data sets, respectively). *H*_o_ and *H*_e_ ranged from 0.034 (QCP) to 0.055 (LB) and from 0.027 (QCP) to 0.064 (MCP), respectively, in the Stacks data set, and from 0.048 (QCP) to 0.065 (MCP) and from 0.035 (QCP) to 0.081 (MCP), respectively, in the ipyrad data set. The values of the inbreeding coefficients (*F*_IS_) were negative in QCP and LBI (Table 3). The genetic summary statistics calculated using the cross-validated SNPs (1,612 SNPs) data sets were basically identical and were in the middle of the values from the Stacks and ipyrad data sets (15,272 and 18,960 SNPs) individually (Supplementary Tables 6, 7).

**TABLE 3:** Summary of genetic diversity and effective population size calculated from two data sets (Stacks data set, ipyrad data set).

Stacks							

Genetic groups	Sampling locations	Samples	*H*_o_	*H*_e_	π	*F*_IS_	*N*_e_ (95%C.I.)
	MCP	15	0.055	0.064	0.067	0.054	59.7 (21.9, inf)
	BSH	12	0.052	0.060	0.063	0.042	39.8 (12.2, inf)
	CEN	15	0.050	0.062	0.064	0.064	16 (4.1, inf)
	JYZ	16	0.050	0.062	0.064	0.064	14.6 (7.4, 39.9)
	LB(LBE)	8	0.053	0.060	0.065	0.032	2.4 (0.8, inf)
HM		66	0.051	0.067	0.068	0.193	74.2 (74.0, 74.4)
LBI	LB(LBI)	7	0.059	0.039	0.042	–0.031	2.0 (0.1, inf)
QSH	QSH	16	0.045	0.054	0.056	0.039	30.7 (10.7, inf)
QCP	QCP	4	0.034	0.027	0.030	–0.005	Inf (1.8, inf)
ALL		**93**	**0.050**	**0.071**	**0.072**	**0.270**	**50.3 (39.1, 66.7)**

**ipyrad**							

**Genetic groups**	**Sampling locations**	**Samples**	***H*_o_**	***H*_e_**	**π**	***F*_IS_**	***N*_e_ (95%C.I.)**

	MCP	15	0.065	0.081	0.084	0.069	38.1 (11.3, inf)
	BSH	12	0.064	0.078	0.082	0.059	48.9 (5.4, inf)
	CEN	15	0.062	0.079	0.083	0.078	15.1 (3.2, inf)
	JYZ	16	0.060	0.080	0.083	0.088	13.3 (6, 44.0)
	LB(LBE)	8	0.063	0.073	0.075	0.043	0.9 (0.5, 1.7)
HM		66	0.063	0.087	0.089	0.215	46.0 (31.0, 76.0)
LBI	LB(LBI)	7	0.063	0.041	0.045	–0.030	1.0 (1.0, 1.0)
QSH	QSH	16	0.052	0.066	0.069	0.051	24.5 (5.2, inf)
QCP	QCP	4	0.048	0.034	0.041	–0.012	inf (0.5, inf)
ALL		**93**	**0.060**	**0.095**	**0.096**	**0.316**	**26.6 (18.5, 39.6)**

*observed heterozygosity (H_o_), expected heterozygosity (H_e_), genetic diversity (π), inbreeding coefficients (F_IS_), contemporary effective population size (N_e_).*

Apart from QCP, where no reliable *N*_e_ estimates could be obtained (infinite effective population size) the *N*_e_ estimated by NeEstimator V2 in the other five sampling locations and two genetic groups in LB ranged from 2.0 (LBI) to 59.7 (MCP) in the Stacks data set and from 0.9 (LBI) to 48.9 (BSH) in the ipyrad data set (Table 3).

### Inference of Demographic History

**FIGURE 4:**
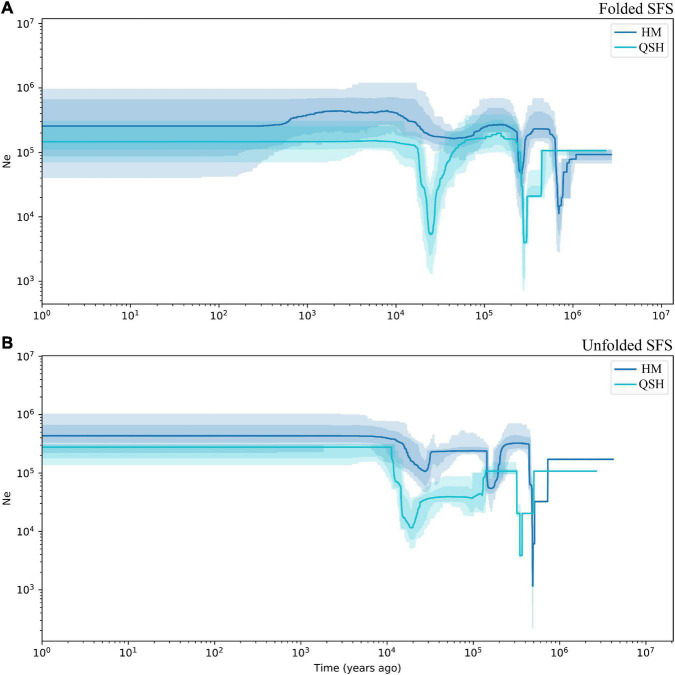
Demographic history of *Magnolia fistulosa* inferred by Stairway plot2. HM and QSH were inferenced with **(A)** folded SFS and **(B)** unfolded SFS. The 95% confidence interval for the estimated effective population size is shown by light color.

Because the very low number of samples in QCP and LBI did not allow accurate inference of demographic history, demographic history was only inferred for all samples, for the HM genetic group and for the QSH genetic group. The demographic history inferred from unfolded SFS was associated with narrower confidence intervals than that inferred from folded SFS (Figure 4 and Supplementary Figure 7). Fluctuations in the effective population size indicated that both populations had experienced three population declines. The earliest decline occurred about 0.5–0.8 Ma ago, the second occurred 0.2–0.3 Ma ago (Figure 4B). During the last glacial stage, the HM genetic group underwent a fluctuation in the effective population size 0.02–0.03 Ma ago, as revealed by unfolded SFS (Figure 4B). In contrast, the folded SFS did not suggest that there was any severe population decline during the last glacial stage (Figure 4A). QSH underwent a bottleneck event in 0.01–0.03 Ma ago, revealed by both folded and unfolded SFS analyses (Figures 4A,B).

## Discussion

### Comparison of the Stacks and Ipyrad Pipelines

The use of different analysis tools can verify the reliability of results (Qi et al., 2015; Boukteb et al., 2021), and the adoption of a reference genome is known to greatly improve the performance of RAD-seq technologies (Torkamaneh et al., 2016). Given that several genomes in the Magnoliaceae were available (BioProject ID PRJNA774088, Dong et al., 2021; Yin et al., 2021), we adopted a reference guided approach to process the population genetics of *Magnolia fistulosa* using both the Stacks and ipyrad pipelines. Although we used the same parameters and filtering criteria, the SNP data set generated by the two pipelines differed in the number of SNPs called and in the average missing rate. Compared to the Stacks pipeline, the genetic statistics calculated by the ipyrad pipeline had higher absolute values. However, despite the differences in the data generated using the two pipelines, the same trends were found in the genetic statistics, population structure analyses and recent gene flow (Supplementary Figures 2–4, 6; Tables 1–3 and Supplementary Tables 5, 7). Groupings inferred via DAPC and PCA analysis are fundamentally the same (Supplementary Figures 2, 4). The Admixture analysis divided QCP and QSH from other samples in most scenarios (Supplementary Figures 8–11).

The choice of analysis method is known to have a large impact on the resulting genotypic information, and to affect the number and quality of the resulting genetic makers (Qi et al., 2015; Torkamaneh et al., 2016). Our results show that the results estimated by the two SNP data sets generated by the reference-based Stacks and ipyrad pipelines are not substantially different.

### Population Structure

The elucidation of the genetic structure of threatened species is paramount to the identification of conservation units (Funk et al., 2012). In our study, QSH and QCP could be separated from other samples from Hekou and Maguan in all of the analyses. However, the samples from Hekou and Maguan showed more complex patterns of population structure. Conflict of population structure inferred using different methods and data sets may indicate the close genetic distances of the samples in Hekou and Maguan (Liu et al., 2020).

Pollen transportation by beetles in *Magnolia* has been proven to be specific, effective, and to make a significant contribution to the cross-pollination (Matsuki et al., 2008; Hernández-Vera et al., 2021). The seeds of *M. fistulosa*, like other *Magnolia* species, are consistent with bird dispersal syndromes (Lomascolo et al., 2008). The low genetic differentiation among individuals in Hekou and Maguan may have benefited from high gene flow facilitated by pollinators and seed dispersal in the past. However, BayesAss analysis revealed low recent gene flow between MCP, BSH, CEN, and JYZ, which may be a result of increased habitat fragmentation caused by the development of farmland in recent decades. Although we cannot say with certainty why the samples from LB comprise two genetic groups, we note that LB is near the Chinese-Vietnamese border, and we, therefore, assume that there are unsampled populations in Vietnam, which have the genetic background seen in LBI (Yang et al., 2015).

The Mantel test of geographic distance and genetic distance between the seven sampling locations indicated a significant pattern of IBD in *Magnolia fistulosa* (Supplementary Figure 6). Additionally, the terrain may also affect the gene flow of this species. The samples in Hekou and Maguan and QSH are found in the Red River valley, while the QCP is found on the other side of the Ailao Mountain, across a phytogeographic boundary known as the Tanaka-Kaiyong Line (Li and Li, 1997; Fan et al., 2013). Although the geographical distance between QSH and QCP is almost the same as that to the HM genetic group (60 km), the former has a greater genetic distance inferred by *F*_*ST*_ (Table 2).

### Genetic Diversity

Small isolated populations often suffer from lower genetic diversity, due to inbreeding, genetic drift and reduced gene flow (Frankham, 2015; Ma et al., 2021b). The four genetic clusters observed in *Magnolia fistulosa* show differences in genetic diversity (Table 3). The HM genetic group, which is the biggest population of *M. fistulosa*, shows highest genetic diversity. QCP, on the contrary, which is the smallest population (four individuals), has the lowest genetic diversity. Although being geographically distant from the other populations, the genetic diversity of QSH was only slightly decreased compared to that of the HM genetic group.

MCP and BSH have relatively large contemporary *N*_e_. MCP comprises more than 70 individuals of *Magnolia fistulosa*, which is the largest locality found to date. We only investigated 20 individuals in BSH, however, there is a further record of *M. fistulosa* in Bojia Village near BSH. Fewer than 20 mature individuals were found in QSH, which is close to the estimated *N*_e_ (30.7 and 24.5). Contemporary *N*_e_ is usually smaller than the real population size (Spies et al., 2020; Zhang et al., 2020), therefore, we believe that there are more, as yet undiscovered, individuals of *M. fistulosa* in QSH. The relatively large contemporary *N*_e_ may explain why the genetic diversity of QSH has not declined significantly (Reed and Frankham, 2003; Frankham et al., 2014). Extremely small contemporary *N*_e_ estimates in both of the two genetic clusters in LB may indicate that the number of individuals involved in reproduction in LB is quite small. The infinite estimates of *N*_*e*_ in the QCP may be due to sampling a limited number of individuals (Do et al., 2014).

*Magnolia fistulosa* has relatively high genetic diversity (calculated to be 0.072 and 0.096 using the Stacks and ipyrad data sets, respectively) compared to some endangered plants assessed using ddRAD-seq. For example, lower genetic diversity was found in *Viola uliginosa* (Violaceae; 0.0440; Lee et al., 2020), *Rhododendron cyanocarpum* (Ericaceae; 0.0702; Liu et al., 2020), *Clermontia fauriei* (Campanulaceae; 0.0014), and *Cyanea pilosa* (Campanulaceae; 0.0012; Jennings et al., 2016). Relatively high genetic diversity and large population differentiation has been found in other threatened *Magnolia* species, including *M. odora* (AFLP analysis, Huang and Zhuang, 2002) and *M. grandis* (ISSR analysis, Chen et al., 2010). The endangered status of these species is therefore likely to have been caused by recent population declines.

### Demographic History of *Magnolia fistulosa*

Inferring the demographic history of endangered species is important for understanding the threats that face them (Yang et al., 2018; Ma et al., 2021b). Stairway plot 2 is a model-flexible method to infer demographic history, and uses SFS calculated from phased or unphased sequence data. As fragmented sequencing has limited influence on the generation of the SFS (Beichman et al., 2018), Stairway plot performs well in studies using RAD-seq technology (Cristofari et al., 2018; Sakaguchi et al., 2021).

The first population decline in *Magnolia fistulosa* is inferred to have occurred about 0.5–0.8 Ma years ago, possibly linked to the Naynayxungla glaciation (0.5–0.8 Ma) (Zheng et al., 2002). The Naynayxungla glaciation was the largest glaciation in the Qinghai-Tibet Plateau during the Middle Pleistocene, and population declines during this period have also been observed in other species in China (Yang et al., 2018; Ma et al., 2021c). A bottleneck in *M. fistulosa* also occurred at about 0.2–0.3 Ma years ago, which is consistent with the time of a high lateral moraine of the penultimate glacial period (316.27 ± 63.2 ka and 257.27 ± 51.4 ka), as dated by electron spin-resonance spectroscopy (Zheng et al., 2002). Temperature drops during this time (0.24–0.28 Ma) have been suggested by many studies (Zheng et al., 2002; Sun and An, 2005). During the last glacial period, the population decline about 0.02–0.03 Ma years ago may correspond to the Last Glacial Maximum (LGM) occurring 19.0–26.5 Ka years ago (Sun and An, 2005; Clark et al., 2009; Zhao et al., 2021).

Although the total *Magnolia fistulosa* population experienced multiple shrinkages in the past (Figure 4 and Supplementary Figure 7), it recovered to a considerably high level after the LGM, which may explain the high genetic diversity we observed. The stable population seen in the recent history may indicate that human disturbance is the most important threat, causing the low current population sizes and restricted distribution of this species.

### Implications for the Conservation and Management of *Magnolia fistulosa*

Despite its small population sizes and narrow distribution, *Magnolia fistulosa* has relatively high genetic diversity and large population differentiation. Based on our findings, we recommend the following conservation management. (1) *Ex situ* conservation process should be carried out to ensure the long-term survival of *M. fistulosa*. Based on the results of our population structure inference, QSH and QCP represent unique genetic resources and should both be collected and protected. Within the samples in Hekou and Maguan, MCP, BSH, and CEN can be considered as one genetic cluster, but the samples from JYZ and LB should be collected separately. (2) Most individuals in the Hekou-Maguan and QSH are already under protection in the Daweishan National Nature Reserve and Gulinqing Provincial Nature Reserve, however, land development may have damaged the connectivity in this area, which is reflected in the low recent gene flow within samples from Hekou and Maguan. To prevent further restriction of gene flow, we recommend restoring some areas of farm land to forest in the Daweishan National Nature Reserve and Gulinqing Provincial Nature Reserve, especially along steams, which are particularly suitable habitats for *M. fistulosa*. (3) Despite the fact that we conducted several investigations, only four individuals were found in the QCP, and these were close together. To prevent further genetic recession, we recommend adopting artificial outcrossing and propagation in this population. Furthermore, because the location of this population does not belong to any nature reserve, we recommend establishing mini-reserves to implement rescue protection of the QCP population (Yang et al., 2020).

## Data Availability Statement

The datasets presented in this study can be found in online repositories. The names of the repository/repositories and accession number(s) can be found below: www.ncbi.nlm.nih.gov/bioproject/PRJNA770186.

## Author Contributions

WS developed the idea. FY and LC designed the experiment, interpreted the results, and wrote the manuscript. FY, LC, and ZD collected the leaf materials. FY performed the statistical analyses. WS and ZD acquired the funding. All authors read and approved the final manuscript.

## Conflict of Interest

The authors declare that the research was conducted in the absence of any commercial or financial relationships that could be construed as a potential conflict of interest.

## Publisher’s Note

All claims expressed in this article are solely those of the authors and do not necessarily represent those of their affiliated organizations, or those of the publisher, the editors and the reviewers. Any product that may be evaluated in this article, or claim that may be made by its manufacturer, is not guaranteed or endorsed by the publisher.
