# Supplementary material for: Genomic Data Reveals Population Genetic and Demographic History of Magnolia fistulosa (Magnoliaceae), a Plant Species With Extremely Small Populations in Yunnan Province, China
Source: Front Plant Sci. 2022 Feb 17;13:811312. doi: 10.3389/fpls.2022.811312 (PMC8892343; doi:10.3389/fpls.2022.811312)
Supplement: Supplementary file 1 [file Data_Sheet_1.docx]

Supplementary Material

**Contents**

**1 Supplementary Figures** 1

Supplementary Figure 1 1

Supplementary Figure 2 1

Supplementary Figure 3 2

Supplementary Figure 4 2

Supplementary Figure 5 3

Supplementary Figure 6 4

Supplementary Figure 7 5

Supplementary Figure 8 6

Supplementary Figure 9 7

Supplementary Figure10 8

Supplementary Figure11 9

**2 Supplementary Tables** 10

Supplementary Table 1 10

Supplementary Table 2 12

Supplementary Table 3 14

Supplementary Table 4 16

Supplementary Table 5 17

Supplementary Table 6 17

Supplementary Table 7 18

# Supplementary Figures


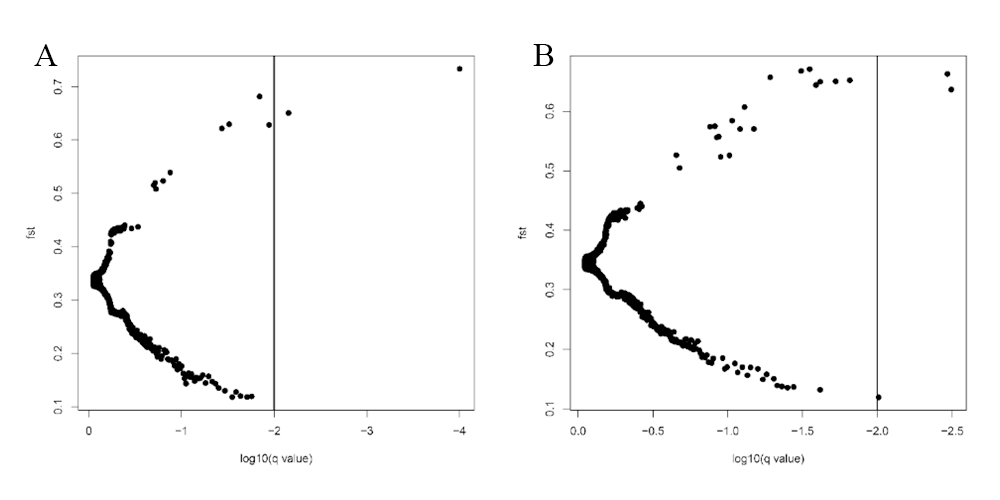


Supplementary Figure 1 Outlier loci detection via Bayescan, where loci with a q-value below 0.01 were considered outlier SNPs, (A) in the Stacks data set and (B) in the ipyrad data set.


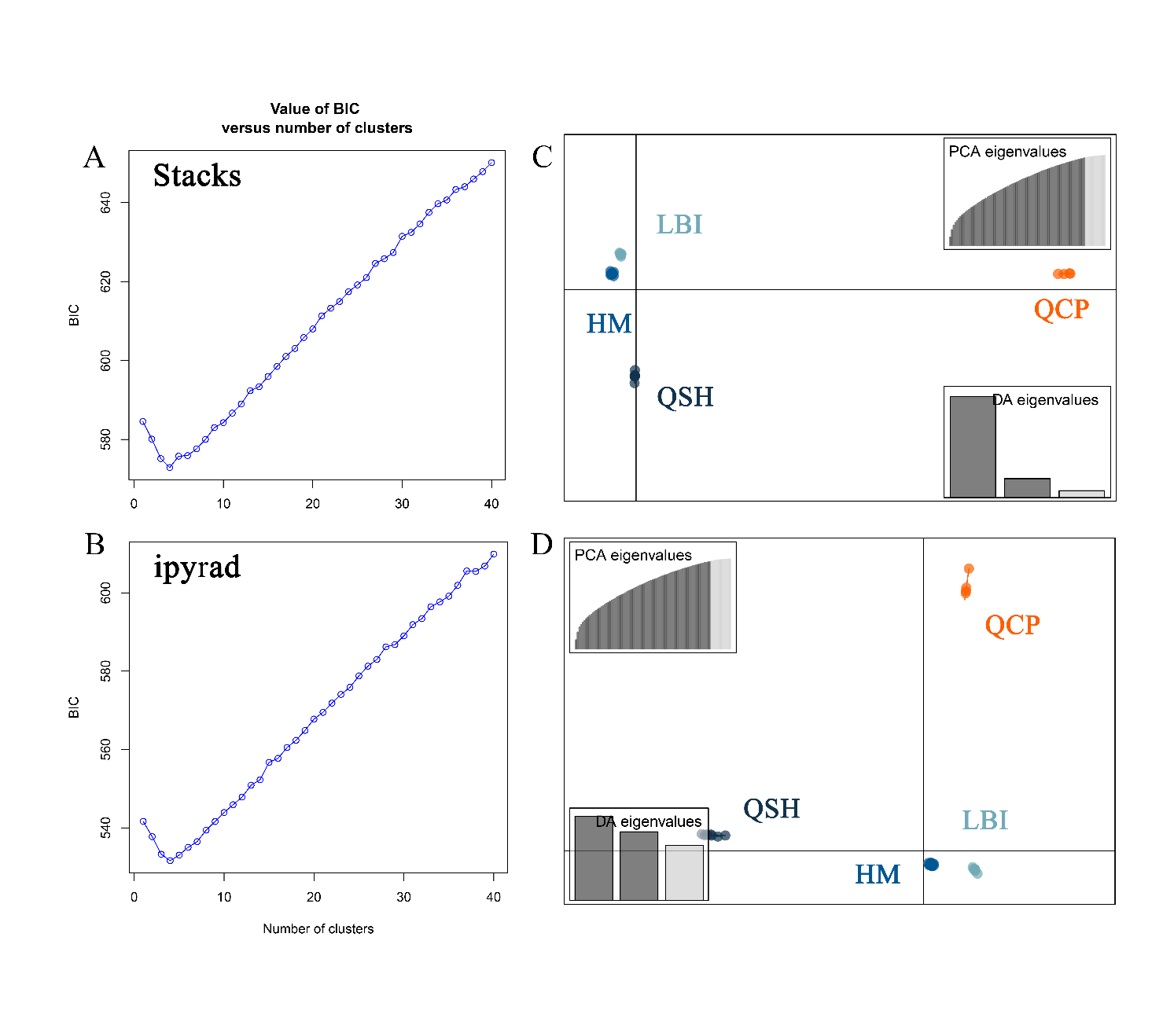


Supplementary Figure 2 BIC values for increasing values of *K* (A) for the Stacks data set and (B) for the ipyrad data set including all samples. DAPC analyses using all samples (C) Stacks data set and (D) ipyrad data set.


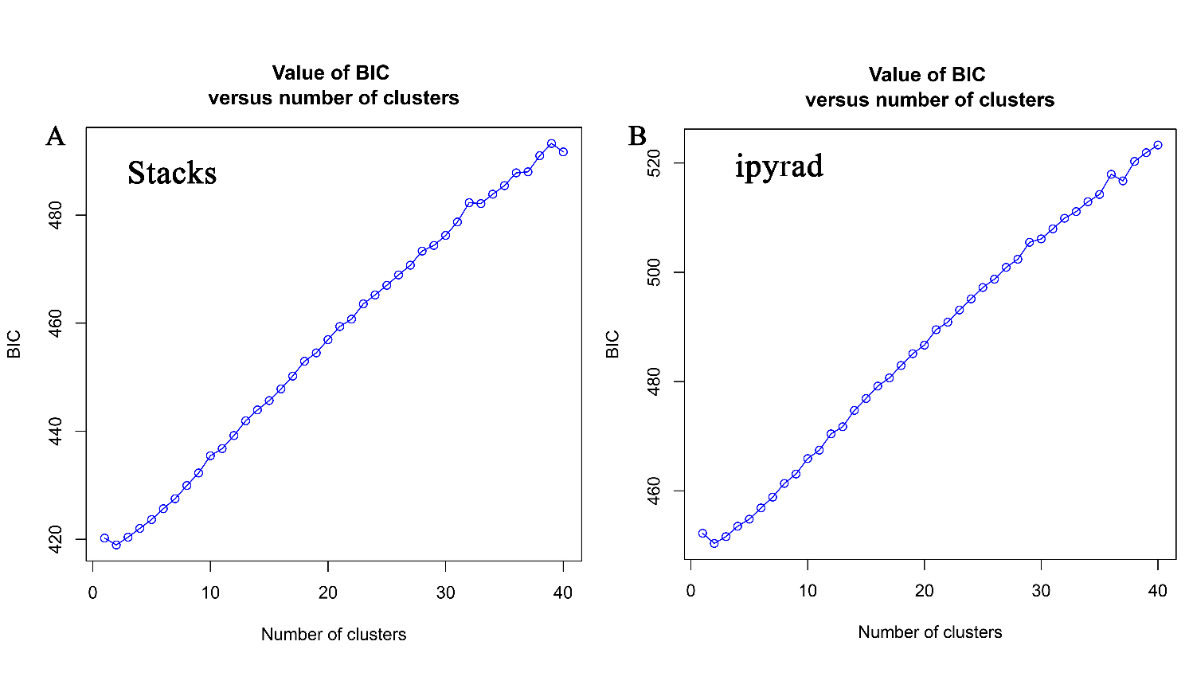


Supplementary Figure 3 BIC values for increasing values of *K* (A) for the Stacks data set and (B) for the ipyrad data set using only samples from Hekou and Maguan.


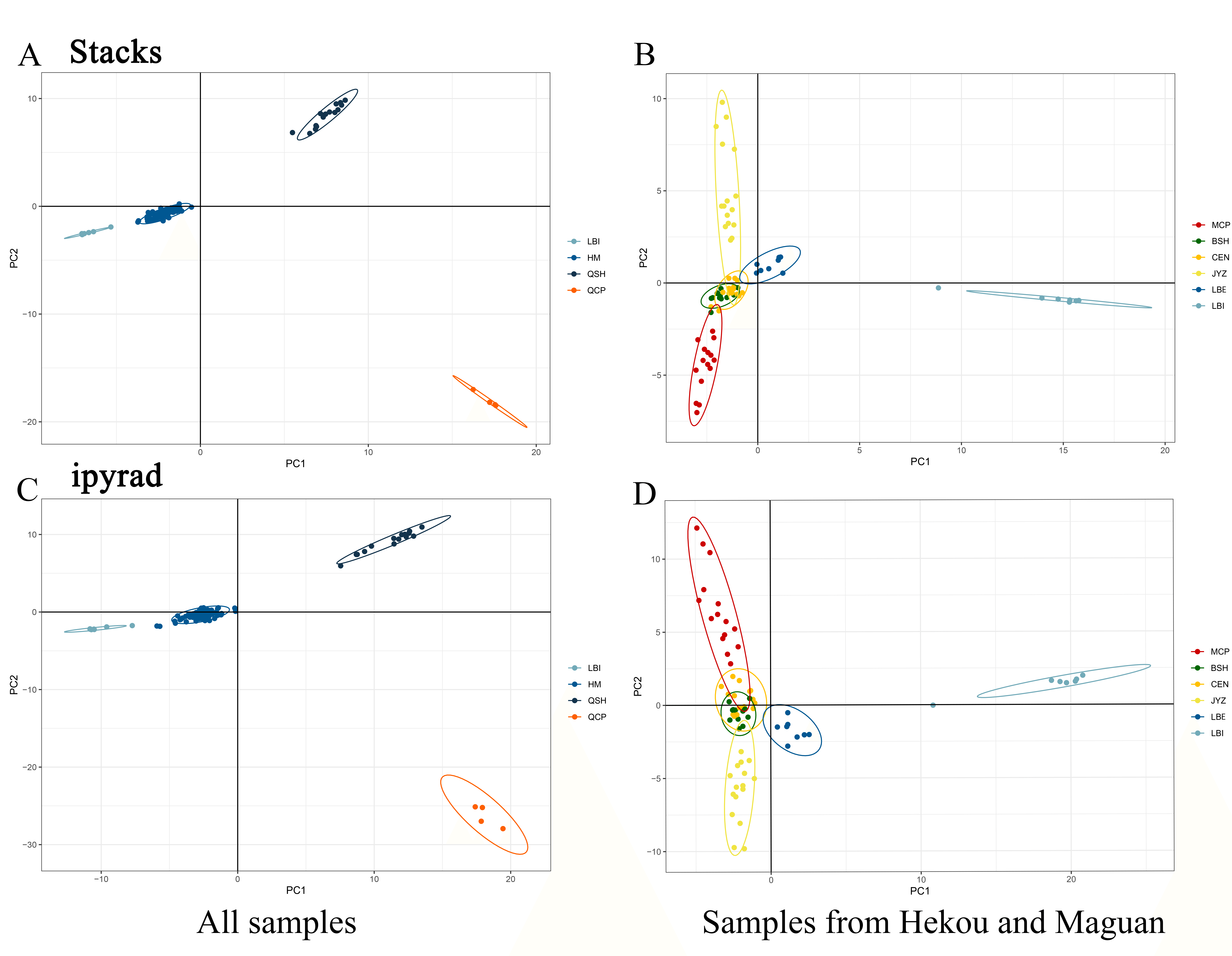


Supplementary Figure 4 PCA analysis using (A, C) all samples or (B, D) only samples from Hekou and Maguan from (A, B) the Stacks data set and (B, D) the ipyrad data set.


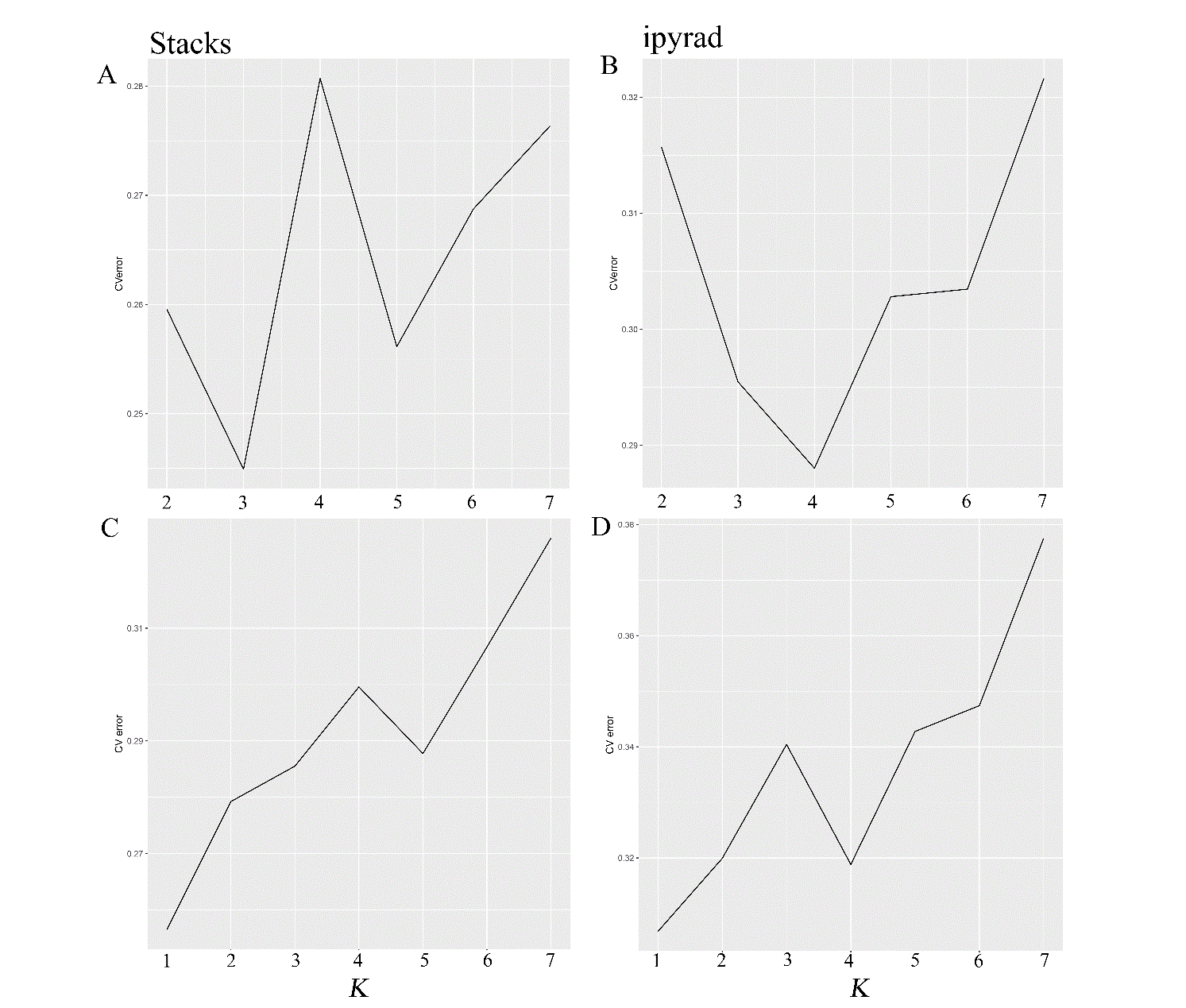


Supplementary Figure 5 The optimal *K* values identified using minimum CV error for (A, B) all samples and (C, D) only for samples from Hekou and Maguan from (A, C) the Stacks data set and (B, D) the ipyrad data set.


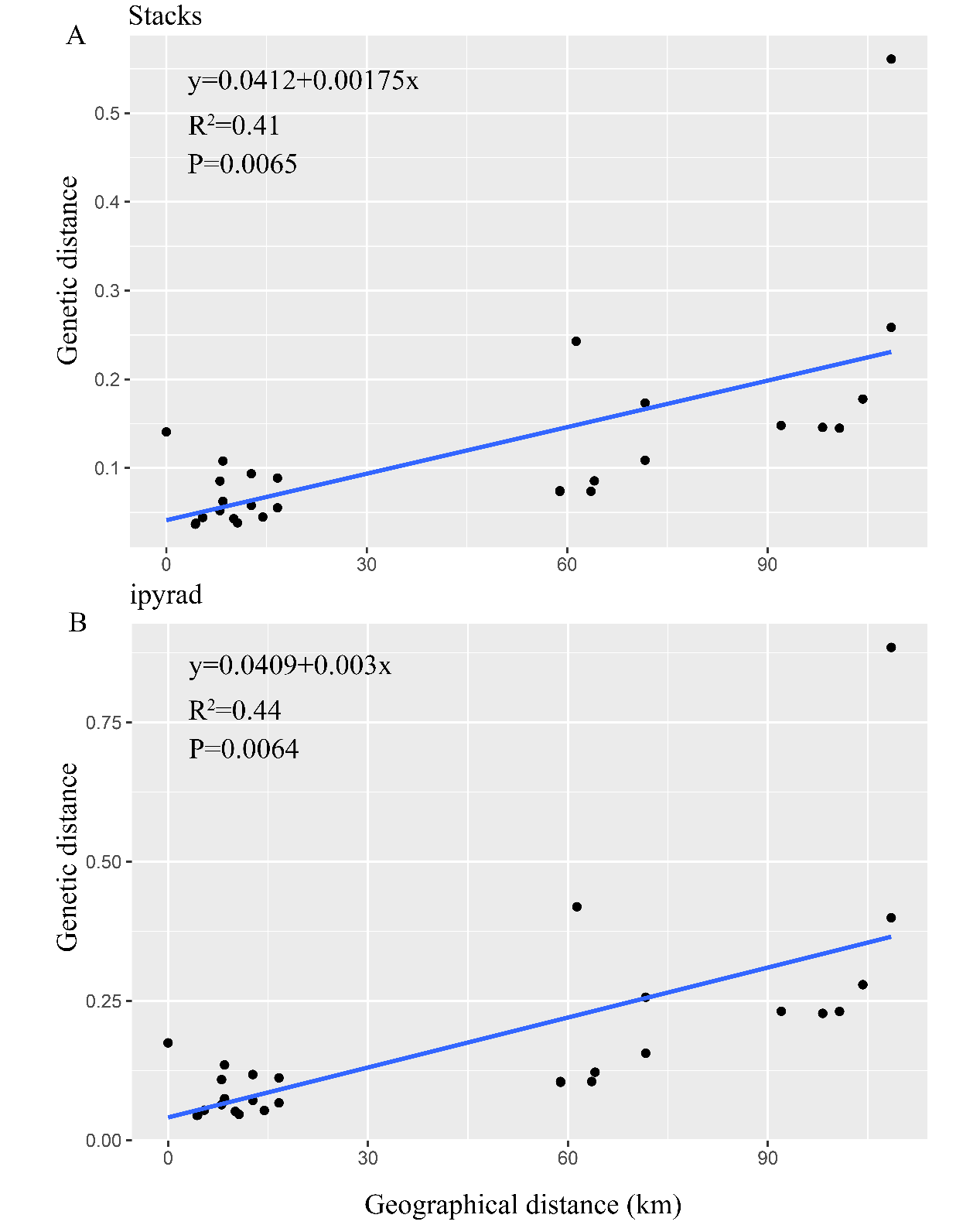


Supplementary Figure 6 Correlations between geographic distance and genetic distance, calculated by *F*_ST_(1- *F*_ST_), for *Magnolia fistulosa* inferred through a Mantel test based (A) on the Stacks data set and (B) on the ipyrad data set.


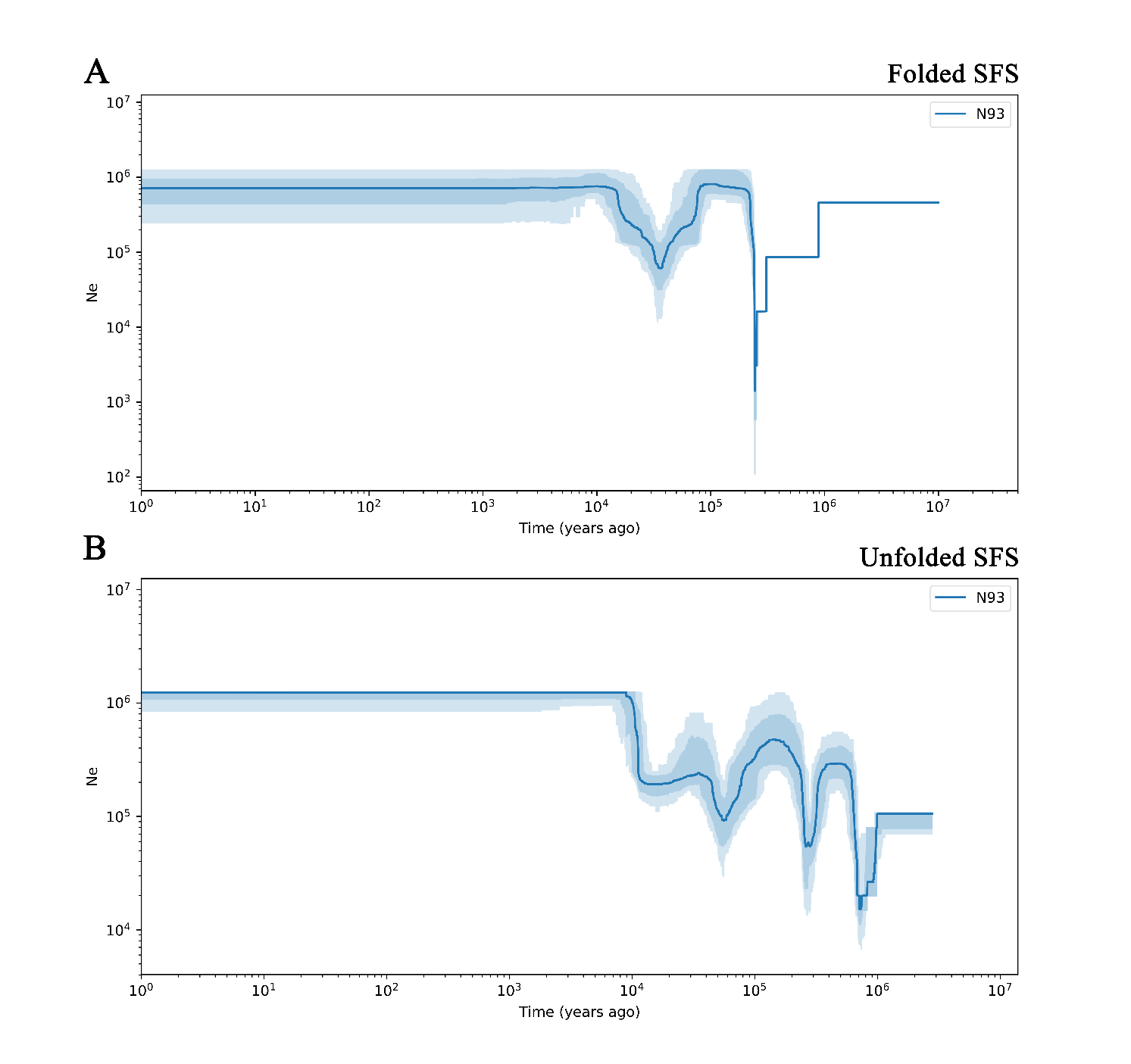


Supplementary Figure 7 Demographic history of *Magnolia fistulosa* inferred using all 93 samples from (A) a folded SFS and from (B) an unfolded SFS.


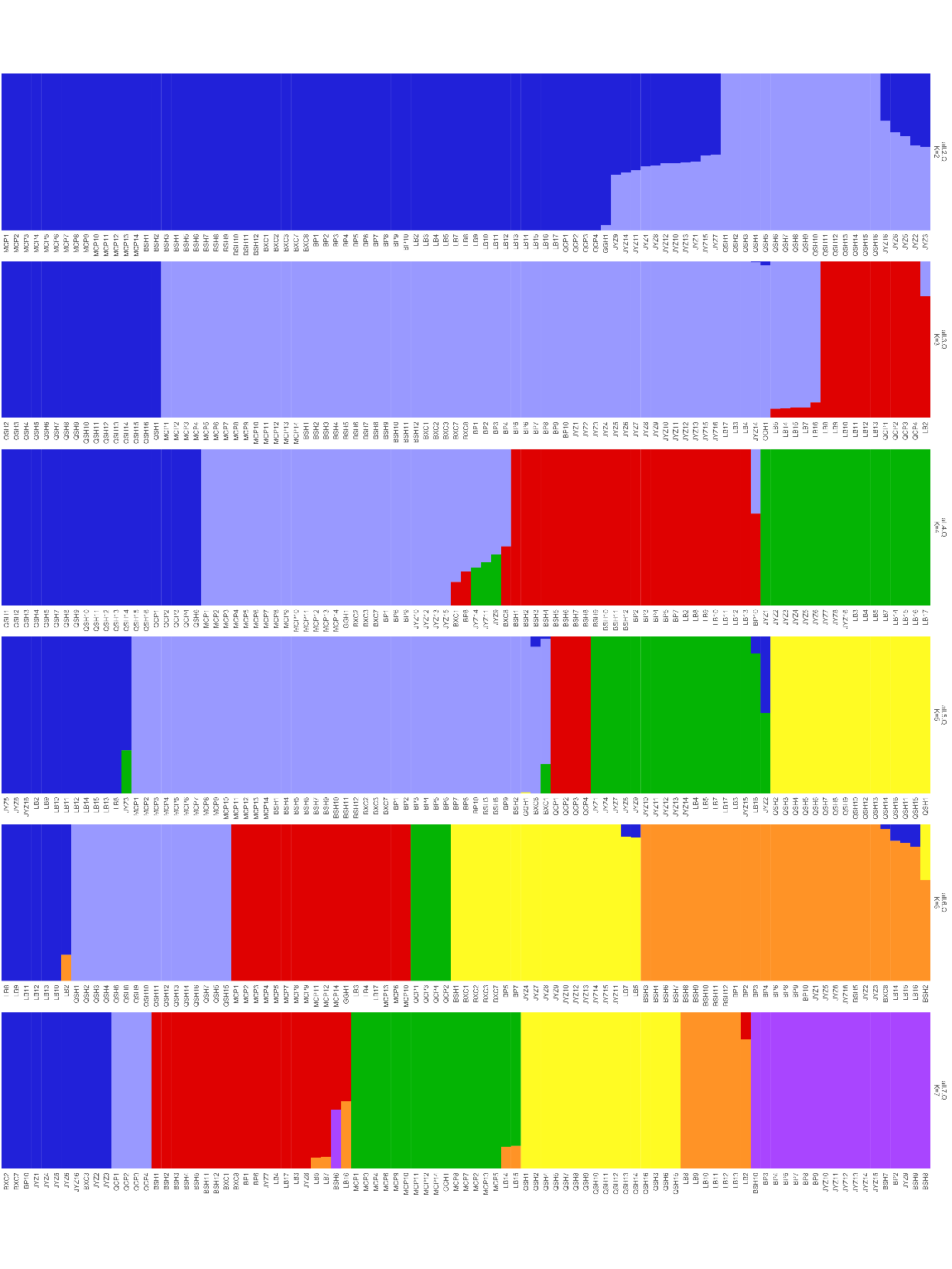


**Supplementary Figure 8** Population structure plots using Stacks data set from all samples with *K* = 2 to *K* = 7.


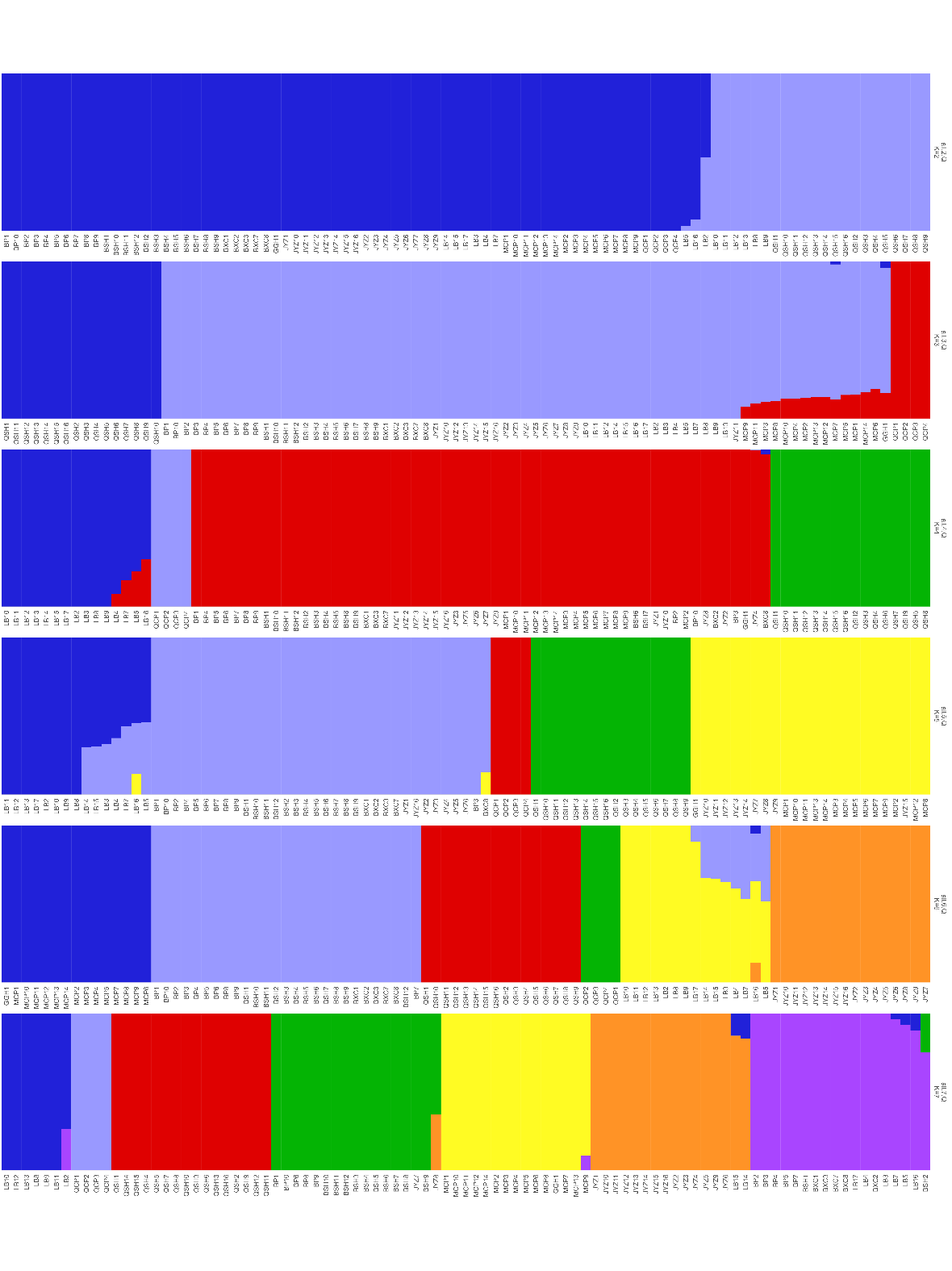


**Supplementary Figure 9** Population structure plots using ipyrad data set from all samples with *K* = 2 to *K* = 7.


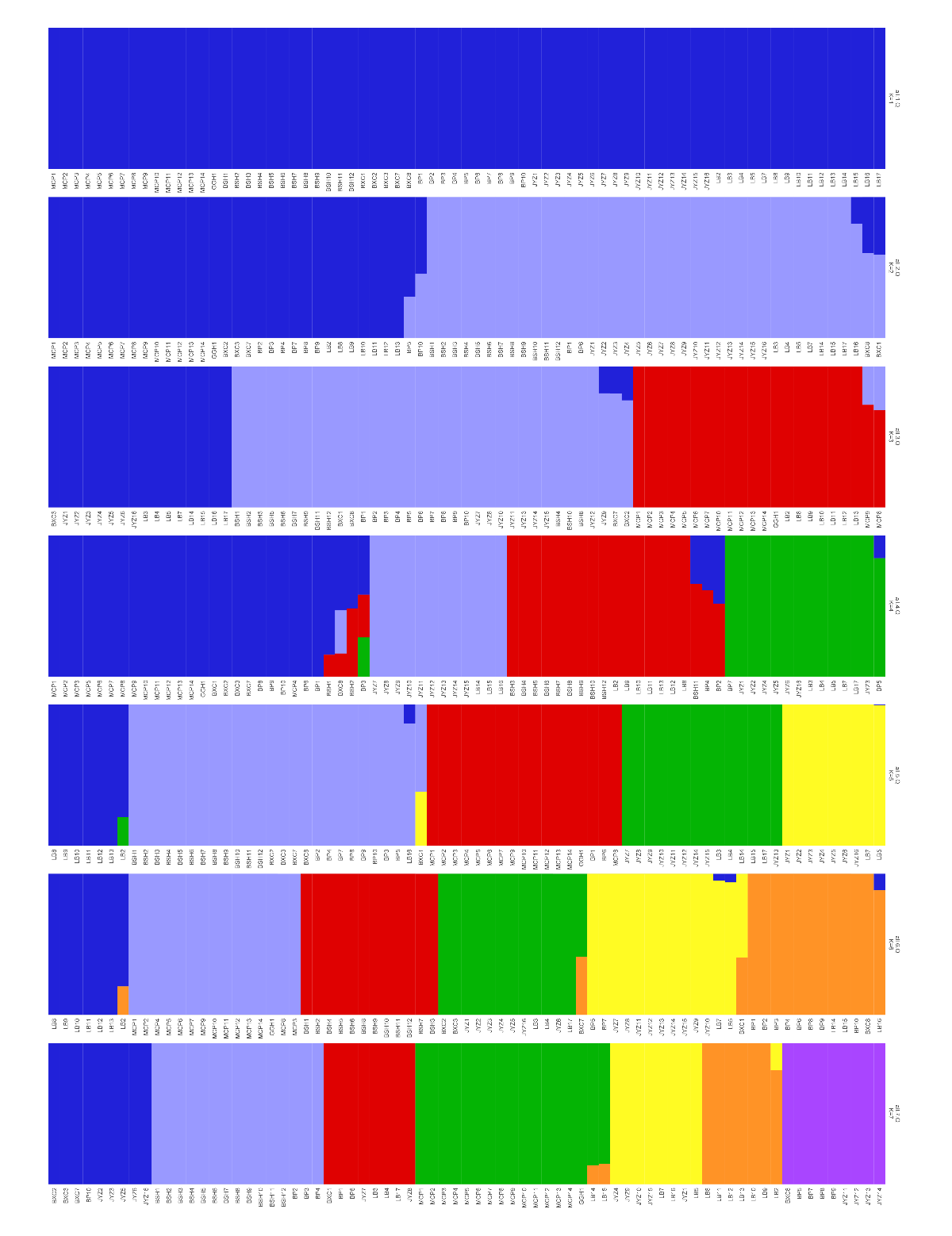


**Supplementary Figure 10** Population structure plots using Stacks data set with the samples from Hekou and Maguan with *K* = 1 to *K* = 7.

.

**
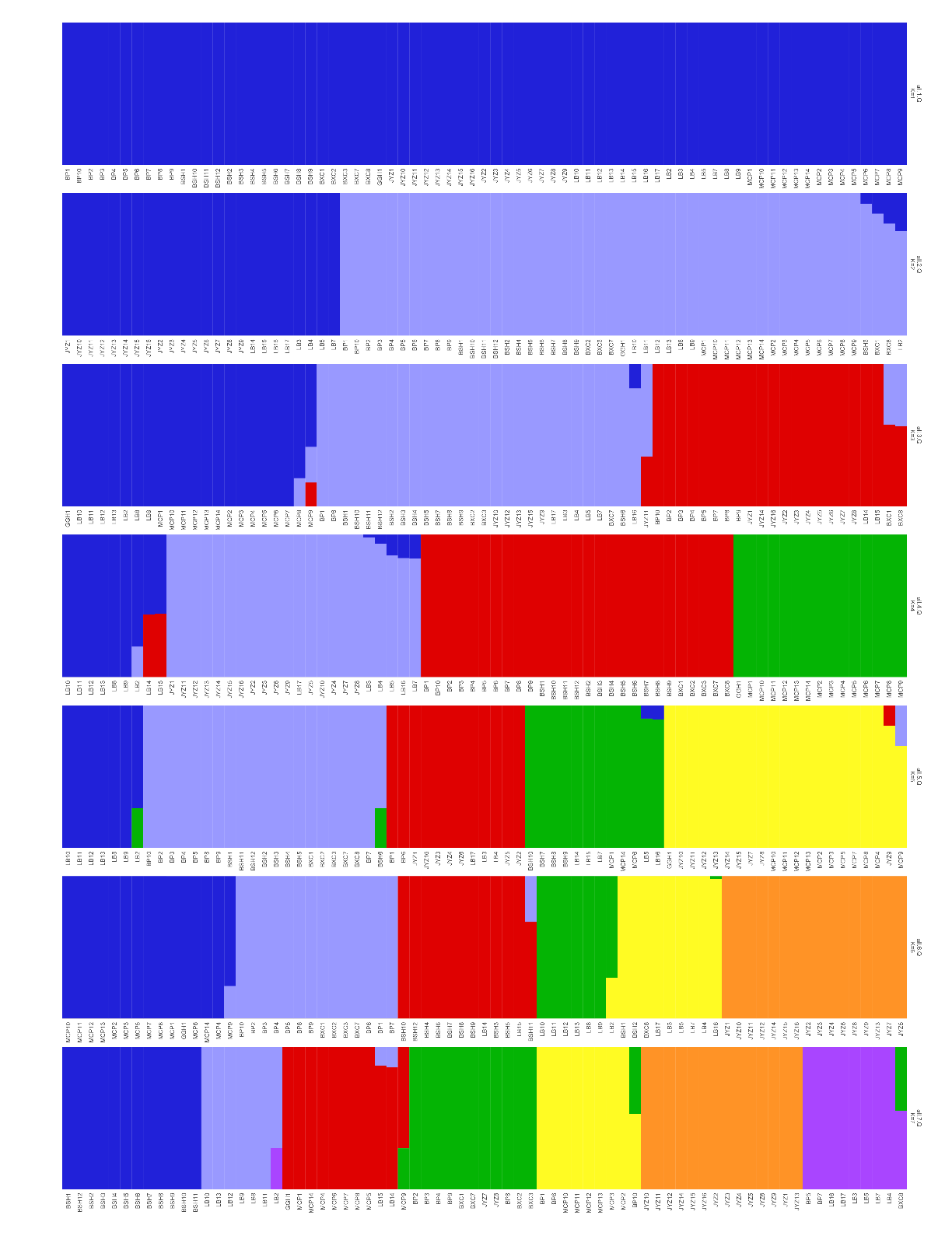
**

**Supplementary Figure 11** Population structure plots using ipyrad data set with the samples in Hekou and Maguan with *K* = 1 to *K* = 7.

# Supplementary Tables

Supplementary Table 1 Summary of filtered and demultiplexed data.

| **Number** | **Sample** | **Raw reads** | **NoRadTag** | **LowQuality** | **Clean reads** | **Mean coverage** |
| --- | --- | --- | --- | --- | --- | --- |
| **1** | MCP1 | 5378372 | 159415 | 7102 | 5211855 | 5.43 |
| **2** | MCP2 | 5827408 | 117250 | 9081 | 5701077 | 5.07 |
| **3** | MCP3 | 2661618 | 80843 | 3636 | 2577139 | 6.79 |
| **4** | MCP4 | 4557120 | 143417 | 7283 | 4406420 | 5.04 |
| **5** | MCP5 | 4518010 | 106613 | 6651 | 4404746 | **4.27** |
| **6** | MCP6 | 4147194 | 82078 | 5953 | 4059163 | 6.17 |
| **7** | MCP7 | 4669564 | 95451 | 5147 | 4568966 | 7.11 |
| **8** | MCP8 | 6148724 | 94340 | 7445 | 6046939 | 4.90 |
| **9** | MCP9 | 4641274 | 92894 | 4920 | 4543460 | 6.84 |
| **10** | MCP10 | 3375424 | 73586 | 4401 | 3297437 | 6.02 |
| **11** | MCP11 | 1897410 | 88774 | 2260 | 1806376 | 8.01 |
| **12** | MCP12 | 3217542 | 76614 | 4242 | 3136686 | 5.10 |
| **13** | MCP13 | 4141352 | 251575 | 748 | 3889029 | 8.10 |
| **14** | MCP14 | 12039104 | 460135 | 2211 | 11576758 | 5.43 |
| **15** | GGH1 | 5356140 | 162908 | 3059 | 5190173 | **12.71** |
| **16** | BSH1 | 3781304 | 137706 | 7720 | 3635878 | 11.47 |
| **17** | BSH2 | 7310794 | 142103 | 12368 | 7156323 | 8.90 |
| **18** | BSH3 | 6899578 | 99611 | 9096 | 6790871 | 6.45 |
| **19** | BSH4 | 4510304 | 90871 | 5595 | 4413838 | 11.05 |
| **20** | BSH5 | 3045042 | 72236 | 4184 | 2968622 | 12.05 |
| **21** | BSH6 | 7198282 | 113577 | 9179 | 7075526 | 6.43 |
| **22** | BSH7 | 5585172 | 117263 | 10914 | 5456995 | 6.10 |
| **23** | BSH8 | 2529596 | 63718 | 3613 | 2462265 | 8.77 |
| **24** | BSH9 | 2733672 | 77647 | 4114 | 2651911 | 7.72 |
| **25** | BSH10 | 1742760 | 63776 | 2862 | 1676122 | 6.35 |
| **26** | BSH11 | 5597826 | 68330 | 6695 | 5522801 | 6.80 |
| **27** | BSH12 | 1958956 | 72124 | 3194 | 1883638 | 8.27 |
| **28** | BXC1 | 5501268 | 121480 | 3289 | 5376499 | 8.42 |
| **29** | BXC2 | 4254702 | 128105 | 5112 | 4121485 | 6.92 |
| **30** | BXC3 | 3293090 | 128199 | 3435 | 3161456 | 8.89 |
| **31** | BXC7 | 3305668 | 109359 | 4724 | 3191585 | 7.35 |
| **32** | BXC8 | 4930658 | 109746 | 4453 | 4816459 | 9.65 |
| **33** | BP1 | 2385520 | 88759 | 3372 | 2293389 | 7.86 |
| **34** | BP2 | 4413824 | 126330 | 5489 | 4282005 | 7.37 |
| **35** | BP3 | 2807336 | 118145 | 5429 | 2683762 | 8.71 |
| **36** | BP4 | 1518172 | 57251 | 2569 | 1458352 | 7.30 |
| **37** | BP5 | 3355782 | 149062 | 5549 | 3201171 | 9.12 |
| **38** | BP6 | 4548692 | 178536 | 6603 | 4363553 | 6.71 |
| **39** | BP7 | 2203674 | 52830 | 2711 | 2148133 | 6.54 |
| **40** | BP8 | 4162014 | 139757 | 4445 | 4017812 | 7.62 |
| **41** | BP9 | 2993878 | 108686 | 4866 | 2880326 | 7.28 |
| **42** | BP10 | 2648824 | 165550 | 4717 | 2478557 | 8.72 |
| **43** | JYZ1 | 3255250 | 99545 | 4801 | 3150904 | 8.00 |
| **44** | JYZ2 | 6944596 | 158698 | 10134 | 6775764 | 9.08 |
| **45** | JYZ3 | 3767884 | 96605 | 5193 | 3666086 | 9.00 |
| **46** | JYZ4 | 3606602 | 211596 | 7206 | 3387800 | 8.94 |
| **47** | JYZ5 | 5176598 | 269855 | 8882 | 4897861 | 8.32 |
| **48** | JYZ6 | 4335674 | 99537 | 6056 | 4230081 | 7.12 |
| **49** | JYZ7 | 5324612 | 208692 | 5878 | 5110042 | 8.58 |
| **50** | JYZ8 | 5989808 | 205170 | 7994 | 5776644 | 7.61 |
| **51** | JYZ9 | 8646020 | 269795 | 11924 | 8364301 | 8.73 |
| **52** | JYZ10 | 8007938 | 181382 | 9516 | 7817040 | 6.03 |
| **53** | JYZ11 | 4863466 | 139716 | 5704 | 4718046 | 6.00 |
| **54** | JYZ12 | 6182732 | 157550 | 7155 | 6018027 | 5.63 |
| **55** | JYZ13 | 5643438 | 258901 | 8965 | 5375572 | 9.50 |
| **56** | JYZ14 | 4673138 | 144587 | 7010 | 4521541 | 9.57 |
| **57** | JYZ15 | 6487428 | 180366 | 8730 | 6298332 | 6.17 |
| **58** | JYZ16 | 3739820 | 42688 | 717 | 3696415 | 8.68 |
| **59** | LB2 | 3983658 | 103477 | 5253 | 3874928 | 7.25 |
| **60** | LB3 | 1810680 | 79272 | 3745 | 1727663 | 7.58 |
| **61** | LB4 | 4977700 | 158062 | 10156 | 4809482 | 4.35 |
| **62** | LB5 | 7405576 | 166058 | 13372 | 7226146 | 6.64 |
| **63** | LB7 | 3170616 | 199964 | 5828 | 2964824 | 6.56 |
| **64** | LB8 | 5686872 | 160526 | 8896 | 5517450 | 4.81 |
| **65** | LB9 | 4545606 | 168485 | 9385 | 4367736 | 6.97 |
| **66** | LB10 | 6523466 | 249126 | 9343 | 6264997 | 6.94 |
| **67** | LB11 | 4875834 | 146268 | 8378 | 4721188 | 8.13 |
| **68** | LB12 | 4977496 | 225439 | 8896 | 4743161 | 6.92 |
| **69** | LB13 | 3716572 | 216791 | 8413 | 3491368 | 9.18 |
| **70** | LB14 | 5030060 | 179117 | 9203 | 4841740 | 8.09 |
| **71** | LB15 | 4599682 | 204376 | 8375 | 4386931 | 10.27 |
| **72** | LB16 | 5312182 | 95910 | 1013 | 5215259 | 8.63 |
| **73** | LB17 | 2490510 | 95096 | 483 | 2394931 | 5.19 |
| **74** | QSH1 | 3990438 | 143162 | 4979 | 3842297 | 7.92 |
| **75** | QSH2 | 2833416 | 88873 | 3396 | 2741147 | 7.23 |
| **76** | QSH3 | 5679132 | 192282 | 7346 | 5479504 | 7.98 |
| **77** | QSH4 | 4794914 | 166347 | 6167 | 4622400 | 5.89 |
| **78** | QSH5 | 7858430 | 172659 | 6077 | 7679694 | 4.54 |
| **79** | QSH6 | 2781778 | 76630 | 3553 | 2701595 | 5.95 |
| **80** | QSH7 | 3505050 | 27185 | 12011 | 3465854 | 7.55 |
| **81** | QSH8 | 8333180 | 27050 | 25592 | 8280538 | 10.13 |
| **82** | QSH9 | 5756522 | 40391 | 18975 | 5697156 | 8.85 |
| **83** | QSH10 | 1881494 | 20023 | 6048 | 1855423 | 5.76 |
| **84** | QSH11 | 3962646 | 28959 | 12342 | 3921345 | 6.92 |
| **85** | QSH12 | 4434948 | 68542 | 886 | 4365520 | 7.36 |
| **86** | QSH13 | 2229398 | 18886 | 7975 | 2202537 | 7.01 |
| **87** | QSH14 | 5895748 | 47287 | 17668 | 5830793 | 7.30 |
| **88** | QSH15 | 4578904 | 44564 | 15317 | 4519023 | 8.93 |
| **89** | QSH16 | 3194430 | 78800 | 601 | 3115029 | 7.15 |
| **90** | QCP1 | 3573572 | 92613 | 3967 | 3476992 | 6.70 |
| **91** | QCP2 | 5766648 | 178512 | 8093 | 5580043 | 8.85 |
| **92** | QCP3 | 3296544 | 115413 | 4722 | 3176409 | 6.67 |
| **93** | QCP4 | 7374206 | 207005 | 9315 | 7157886 | 11.17 |
| **Summary** |  | **425265556** | **11994483** | **622070** | **412649003** |  |
| **Average** |  |  |  |  |  | **7.49** |

Supplementary Table 2 Mapping rates of each sample to each of the three genomes.

| ***Magnolia sinica*** | **Mapping rates** | ***Magnolia officinalis*** | **Mapping rates** | ***Magnolia biondii*** | **Mapping rates** |
| --- | --- | --- | --- | --- | --- |
| MCP1 | 87.18% | MCP1 | 85.77% | MCP1 | 85.94% |
| MCP2 | 86.72% | MCP2 | 85.42% | MCP2 | 85.62% |
| MCP3 | 84.10% | MCP3 | 83.18% | MCP3 | 83.14% |
| MCP4 | 85.88% | MCP4 | 84.64% | MCP4 | 84.74% |
| MCP5 | 86.42% | MCP5 | 85.30% | MCP5 | 85.37% |
| MCP6 | 83.50% | MCP6 | 82.36% | MCP6 | 82.50% |
| MCP7 | 82.29% | MCP7 | 81.03% | MCP7 | 81.21% |
| MCP8 | 86.16% | MCP8 | 85.17% | MCP8 | 85.17% |
| MCP9 | 86.69% | MCP9 | 85.51% | MCP9 | 85.63% |
| MCP10 | 86.87% | MCP10 | 85.88% | MCP10 | 85.93% |
| MCP11 | 85.88% | MCP11 | 85.65% | MCP11 | 85.72% |
| MCP12 | 83.92% | MCP12 | 84.74% | MCP12 | 84.71% |
| MCP13 | 88.65% | MCP13 | 82.70% | MCP13 | 82.69% |
| MCP14 | 87.10% | MCP14 | 87.26% | MCP14 | 87.40% |
| GGH1 | 83.42% | GGH1 | 82.32% | GGH1 | 82.18% |
| BSH1 | 87.91% | BSH1 | 86.59% | BSH1 | 86.93% |
| BSH2 | 89.68% | BSH2 | 88.03% | BSH2 | 88.39% |
| BSH3 | 91.04% | BSH3 | 89.51% | BSH3 | 89.80% |
| BSH4 | 88.02% | BSH4 | 86.47% | BSH4 | 86.86% |
| BSH5 | 86.83% | BSH5 | 85.28% | BSH5 | 85.55% |
| BSH6 | 89.90% | BSH6 | 88.39% | BSH6 | 88.67% |
| BSH7 | 89.90% | BSH7 | 87.97% | BSH7 | 88.19% |
| BSH8 | 89.05% | BSH8 | 87.61% | BSH8 | 88.00% |
| BSH9 | 87.05% | BSH9 | 85.68% | BSH9 | 86.07% |
| BSH10 | 86.16% | BSH10 | 86.57% | BSH10 | 86.93% |
| BSH11 | 83.00% | BSH11 | 84.50% | BSH11 | 84.80% |
| BSH12 | 88.16% | BSH12 | 82.18% | BSH12 | 82.27% |
| BXC1 | 86.08% | BXC1 | 85.18% | BXC1 | 85.16% |
| BXC2 | 82.66% | BXC2 | 82.24% | BXC2 | 81.50% |
| BXC3 | 83.71% | BXC3 | 83.17% | BXC3 | 82.76% |
| BXC7 | 85.38% | BXC7 | 84.14% | BXC7 | 84.13% |
| BXC8 | 85.42% | BXC8 | 84.67% | BXC8 | 84.53% |
| BP1 | 85.47% | BP1 | 82.63% | BP1 | 82.74% |
| BP2 | 85.64% | BP2 | 84.58% | BP2 | 84.67% |
| BP3 | 82.82% | BP3 | 82.18% | BP3 | 82.30% |
| BP4 | 84.97% | BP4 | 84.00% | BP4 | 84.25% |
| BP5 | 83.84% | BP5 | 82.50% | BP5 | 82.75% |
| BP6 | 83.98% | BP6 | 83.12% | BP6 | 83.32% |
| BP7 | 84.24% | BP7 | 83.42% | BP7 | 83.53% |
| BP8 | 81.47% | BP8 | 80.44% | BP8 | 80.62% |
| BP9 | 85.59% | BP9 | 84.69% | BP9 | 84.71% |
| BP10 | 83.91% | BP10 | 84.78% | BP10 | 84.91% |
| JYZ1 | 83.78% | JYZ1 | 83.14% | JYZ1 | 83.25% |
| JYZ2 | 85.63% | JYZ2 | 84.52% | JYZ2 | 84.76% |
| JYZ3 | 87.55% | JYZ3 | 86.24% | JYZ3 | 86.55% |
| JYZ4 | 83.47% | JYZ4 | 82.36% | JYZ4 | 82.62% |
| JYZ5 | 84.84% | JYZ5 | 83.89% | JYZ5 | 84.12% |
| JYZ6 | 86.57% | JYZ6 | 85.38% | JYZ6 | 85.67% |
| JYZ7 | 80.82% | JYZ7 | 79.90% | JYZ7 | 80.23% |
| JYZ8 | 87.36% | JYZ8 | 86.20% | JYZ8 | 86.33% |
| JYZ9 | 87.70% | JYZ9 | 86.53% | JYZ9 | 86.80% |
| JYZ10 | 84.94% | JYZ10 | 82.65% | JYZ10 | 82.92% |
| JYZ11 | 86.46% | JYZ11 | 84.06% | JYZ11 | 84.24% |
| JYZ12 | 87.08% | JYZ12 | 85.55% | JYZ12 | 85.89% |
| JYZ13 | 86.28% | JYZ13 | 85.96% | JYZ13 | 86.11% |
| JYZ14 | 87.38% | JYZ14 | 85.41% | JYZ14 | 85.46% |
| JYZ15 | 84.39% | JYZ15 | 86.26% | JYZ15 | 86.48% |
| JYZ16 | 84.01% | JYZ16 | 83.00% | JYZ16 | 83.11% |
| LB2 | 85.57% | LB2 | 84.47% | LB2 | 84.64% |
| LB3 | 85.96% | LB3 | 84.67% | LB3 | 84.95% |
| LB4 | 87.19% | LB4 | 85.32% | LB4 | 85.70% |
| LB5 | 88.08% | LB5 | 86.37% | LB5 | 86.77% |
| LB7 | 86.01% | LB7 | 84.52% | LB7 | 84.85% |
| LB8 | 86.59% | LB8 | 84.97% | LB8 | 85.37% |
| LB9 | 87.27% | LB9 | 85.73% | LB9 | 86.05% |
| LB10 | 86.66% | LB10 | 85.10% | LB10 | 85.47% |
| LB11 | 87.08% | LB11 | 85.82% | LB11 | 86.10% |
| LB12 | 85.69% | LB12 | 84.23% | LB12 | 84.62% |
| LB13 | 85.01% | LB13 | 83.71% | LB13 | 83.97% |
| LB14 | 86.60% | LB14 | 84.99% | LB14 | 85.36% |
| LB15 | 86.09% | LB15 | 84.42% | LB15 | 84.78% |
| LB16 | 87.15% | LB16 | 85.82% | LB16 | 85.91% |
| LB17 | 86.64% | LB17 | 85.32% | LB17 | 85.54% |
| QSH1 | 88.90% | QSH1 | 82.92% | QSH1 | 82.97% |
| QSH2 | 79.86% | QSH2 | 78.80% | QSH2 | 78.74% |
| QSH3 | 85.15% | QSH3 | 84.59% | QSH3 | 84.51% |
| QSH4 | 85.38% | QSH4 | 84.79% | QSH4 | 84.65% |
| QSH5 | 84.00% | QSH5 | 83.19% | QSH5 | 83.13% |
| QSH6 | 83.51% | QSH6 | 82.80% | QSH6 | 82.63% |
| QSH7 | 89.74% | QSH7 | 87.89% | QSH7 | 88.27% |
| QSH8 | 90.52% | QSH8 | 88.56% | QSH8 | 88.91% |
| QSH9 | 89.16% | QSH9 | 87.47% | QSH9 | 87.88% |
| QSH10 | 90.12% | QSH10 | 87.10% | QSH10 | 87.49% |
| QSH11 | 89.29% | QSH11 | 87.65% | QSH11 | 88.05% |
| QSH12 | 89.14% | QSH12 | 87.95% | QSH12 | 88.12% |
| QSH13 | 89.32% | QSH13 | 87.09% | QSH13 | 87.52% |
| QSH14 | 89.77% | QSH14 | 87.52% | QSH14 | 87.98% |
| QSH15 | 86.70% | QSH15 | 87.50% | QSH15 | 87.96% |
| QSH16 | 83.56% | QSH16 | 84.52% | QSH16 | 84.85% |
| QCP1 | 83.80% | QCP1 | 83.11% | QCP1 | 83.16% |
| QCP2 | 78.80% | QCP2 | 77.87% | QCP2 | 77.94% |
| QCP3 | 82.75% | QCP3 | 82.55% | QCP3 | 81.67% |
| QCP4 | 86.46% | QCP4 | 85.36% | QCP4 | 85.49% |
| **Average** | **86.00%** |  | **84.77%** |  | **84.93%** |

Supplementary Table 3 Missing rates of each sample in two data sets (Stacks data set, ipyrad data set).

| **Ipyrad** |  |  |  |  | **Stacks** |  |  |  |
| --- | --- | --- | --- | --- | --- | --- | --- | --- |
| **Sample** | **N_DATA** | **Missing sites** | **Missing rate** |  | **Sample** | **N_DATA** | **Missing sites** | **Missing rate** |
| BP1 | 18963 | 3494 | 0.184 |  | MCP1 | 15274 | 267 | 0.017 |
| BP10 | 18963 | 4802 | 0.253 |  | MCP2 | 15274 | 271 | 0.018 |
| BP2 | 18963 | 1051 | 0.055 |  | MCP3 | 15274 | 1062 | 0.070 |
| BP3 | 18963 | 4339 | 0.229 |  | MCP4 | 15274 | 451 | 0.030 |
| BP4 | 18963 | 8518 | 0.449 |  | MCP5 | 15274 | 362 | 0.024 |
| BP5 | 18963 | 1962 | 0.103 |  | MCP6 | 15274 | 471 | 0.031 |
| BP6 | 18963 | 934 | 0.049 |  | MCP7 | 15274 | 388 | 0.025 |
| BP7 | 18963 | 4662 | 0.246 |  | MCP8 | 15274 | 274 | 0.018 |
| BP8 | 18963 | 1210 | 0.064 |  | MCP9 | 15274 | 345 | 0.023 |
| BP9 | 18963 | 3530 | 0.186 |  | MCP10 | 15274 | 462 | 0.030 |
| BSH1 | 18963 | 1732 | 0.091 |  | MCP11 | 15274 | 1541 | 0.101 |
| BSH10 | 18963 | 6579 | 0.347 |  | MCP12 | 15274 | 578 | 0.038 |
| BSH11 | 18963 | 967 | 0.051 |  | MCP13 | 15274 | 2373 | 0.155 |
| BSH12 | 18963 | 8357 | 0.441 |  | MCP14 | 15274 | 404 | 0.026 |
| BSH2 | 18963 | 823 | 0.043 |  | GGH1 | 15274 | 1146 | 0.075 |
| BSH3 | 18963 | 1011 | 0.053 |  | BSH1 | 15274 | 461 | 0.030 |
| BSH4 | 18963 | 1016 | 0.054 |  | BSH2 | 15274 | 211 | 0.014 |
| BSH5 | 18963 | 1892 | 0.100 |  | BSH3 | 15274 | 300 | 0.020 |
| BSH6 | 18963 | 734 | 0.039 |  | BSH4 | 15274 | 378 | 0.025 |
| BSH7 | 18963 | 1867 | 0.098 |  | BSH5 | 15274 | 587 | 0.038 |
| BSH8 | 18963 | 4007 | 0.211 |  | BSH6 | 15274 | 209 | 0.014 |
| BSH9 | 18963 | 2855 | 0.151 |  | BSH7 | 15274 | 660 | 0.043 |
| BXC1 | 18963 | 1236 | 0.065 |  | BSH8 | 15274 | 988 | 0.065 |
| BXC2 | 18963 | 2925 | 0.154 |  | BSH9 | 15274 | 711 | 0.047 |
| BXC3 | 18963 | 3669 | 0.193 |  | BSH10 | 15274 | 1978 | 0.130 |
| BXC7 | 18963 | 2232 | 0.118 |  | BSH11 | 15274 | 301 | 0.020 |
| BXC8 | 18963 | 1300 | 0.069 |  | BSH12 | 15274 | 2697 | 0.177 |
| GGH1 | 18963 | 2841 | 0.150 |  | BXC1 | 15274 | 570 | 0.037 |
| JYZ1 | 18963 | 4205 | 0.222 |  | BXC2 | 15274 | 1089 | 0.071 |
| JYZ10 | 18963 | 809 | 0.043 |  | BXC3 | 15274 | 1584 | 0.104 |
| JYZ11 | 18963 | 1978 | 0.104 |  | BXC7 | 15274 | 916 | 0.060 |
| JYZ12 | 18963 | 1357 | 0.072 |  | BXC8 | 15274 | 469 | 0.031 |
| JYZ13 | 18963 | 1414 | 0.075 |  | BP1 | 15274 | 868 | 0.057 |
| JYZ14 | 18963 | 1878 | 0.099 |  | BP2 | 15274 | 368 | 0.024 |
| JYZ15 | 18963 | 1122 | 0.059 |  | BP3 | 15274 | 1314 | 0.086 |
| JYZ16 | 18963 | 5628 | 0.297 |  | BP4 | 15274 | 2747 | 0.180 |
| JYZ2 | 18963 | 1121 | 0.059 |  | BP5 | 15274 | 537 | 0.035 |
| JYZ3 | 18963 | 2634 | 0.139 |  | BP6 | 15274 | 359 | 0.024 |
| JYZ4 | 18963 | 4185 | 0.221 |  | BP7 | 15274 | 1231 | 0.081 |
| JYZ5 | 18963 | 1959 | 0.103 |  | BP8 | 15274 | 453 | 0.030 |
| JYZ6 | 18963 | 1898 | 0.100 |  | BP9 | 15274 | 985 | 0.064 |
| JYZ7 | 18963 | 4660 | 0.246 |  | BP10 | 15274 | 1422 | 0.093 |
| JYZ8 | 18963 | 1314 | 0.069 |  | JYZ1 | 15274 | 1188 | 0.078 |
| JYZ9 | 18963 | 558 | 0.029 |  | JYZ2 | 15274 | 368 | 0.024 |
| LB10 | 18963 | 675 | 0.036 |  | JYZ3 | 15274 | 706 | 0.046 |
| LB11 | 18963 | 924 | 0.049 |  | JYZ4 | 15274 | 1386 | 0.091 |
| LB12 | 18963 | 752 | 0.040 |  | JYZ5 | 15274 | 597 | 0.039 |
| LB13 | 18963 | 2550 | 0.134 |  | JYZ6 | 15274 | 641 | 0.042 |
| LB14 | 18963 | 792 | 0.042 |  | JYZ7 | 15274 | 1444 | 0.095 |
| LB15 | 18963 | 1135 | 0.060 |  | JYZ8 | 15274 | 400 | 0.026 |
| LB16 | 18963 | 4554 | 0.240 |  | JYZ9 | 15274 | 235 | 0.015 |
| LB17 | 18963 | 10291 | 0.543 |  | JYZ10 | 15274 | 258 | 0.017 |
| LB2 | 18963 | 1214 | 0.064 |  | JYZ11 | 15274 | 631 | 0.041 |
| LB3 | 18963 | 6136 | 0.324 |  | JYZ12 | 15274 | 416 | 0.027 |
| LB4 | 18963 | 944 | 0.050 |  | JYZ13 | 15274 | 448 | 0.029 |
| LB5 | 18963 | 631 | 0.033 |  | JYZ14 | 15274 | 575 | 0.038 |
| LB7 | 18963 | 1935 | 0.102 |  | JYZ15 | 15274 | 403 | 0.026 |
| LB8 | 18963 | 434 | 0.023 |  | JYZ16 | 15274 | 2244 | 0.147 |
| LB9 | 18963 | 1457 | 0.077 |  | LB2 | 15274 | 401 | 0.026 |
| QSH1 | 18963 | 2558 | 0.135 |  | LB3 | 15274 | 2141 | 0.140 |
| QSH10 | 18963 | 7805 | 0.412 |  | LB4 | 15274 | 416 | 0.027 |
| QSH11 | 18963 | 2532 | 0.134 |  | LB5 | 15274 | 237 | 0.016 |
| QSH12 | 18963 | 7024 | 0.370 |  | LB7 | 15274 | 712 | 0.047 |
| QSH13 | 18963 | 5672 | 0.299 |  | LB8 | 15274 | 172 | 0.011 |
| QSH14 | 18963 | 1549 | 0.082 |  | LB9 | 15274 | 438 | 0.029 |
| QSH15 | 18963 | 2118 | 0.112 |  | LB10 | 15274 | 174 | 0.011 |
| QSH16 | 18963 | 5499 | 0.290 |  | LB11 | 15274 | 296 | 0.019 |
| QSH2 | 18963 | 6397 | 0.337 |  | LB12 | 15274 | 261 | 0.017 |
| QSH3 | 18963 | 1525 | 0.080 |  | LB13 | 15274 | 765 | 0.050 |
| QSH4 | 18963 | 1820 | 0.096 |  | LB14 | 15274 | 278 | 0.018 |
| QSH5 | 18963 | 1937 | 0.102 |  | LB15 | 15274 | 374 | 0.024 |
| QSH6 | 18963 | 6422 | 0.339 |  | LB16 | 15274 | 1476 | 0.097 |
| QSH7 | 18963 | 3502 | 0.185 |  | LB17 | 15274 | 4709 | 0.308 |
| QSH8 | 18963 | 1086 | 0.057 |  | QSH1 | 15274 | 972 | 0.064 |
| QSH9 | 18963 | 1738 | 0.092 |  | QSH2 | 15274 | 2844 | 0.186 |
| MCP1 | 18963 | 641 | 0.034 |  | QSH3 | 15274 | 475 | 0.031 |
| MCP10 | 18963 | 1554 | 0.082 |  | QSH4 | 15274 | 720 | 0.047 |
| MCP11 | 18963 | 5207 | 0.275 |  | QSH5 | 15274 | 449 | 0.029 |
| MCP12 | 18963 | 2068 | 0.109 |  | QSH6 | 15274 | 3094 | 0.203 |
| MCP13 | 18963 | 6508 | 0.343 |  | QSH7 | 15274 | 662 | 0.043 |
| MCP14 | 18963 | 1986 | 0.105 |  | QSH8 | 15274 | 205 | 0.013 |
| MCP2 | 18963 | 582 | 0.031 |  | QSH9 | 15274 | 300 | 0.020 |
| MCP3 | 18963 | 3482 | 0.184 |  | QSH10 | 15274 | 1497 | 0.098 |
| MCP4 | 18963 | 1307 | 0.069 |  | QSH11 | 15274 | 457 | 0.030 |
| MCP5 | 18963 | 906 | 0.048 |  | QSH12 | 15274 | 2683 | 0.176 |
| MCP6 | 18963 | 1335 | 0.070 |  | QSH13 | 15274 | 992 | 0.065 |
| MCP7 | 18963 | 864 | 0.046 |  | QSH14 | 15274 | 316 | 0.021 |
| MCP8 | 18963 | 683 | 0.036 |  | QSH15 | 15274 | 503 | 0.033 |
| MCP9 | 18963 | 869 | 0.046 |  | QSH16 | 15274 | 2385 | 0.156 |
| QCP1 | 18963 | 3562 | 0.188 |  | QCP1 | 15274 | 0 | 0.000 |
| QCP2 | 18963 | 1939 | 0.102 |  | QCP2 | 15274 | 0 | 0.000 |
| QCP3 | 18963 | 4529 | 0.239 |  | QCP3 | 15274 | 0 | 0.000 |
| QCP4 | 18963 | 1290 | 0.068 |  | QCP4 | 15274 | 0 | 0.000 |
| **Average** |  |  | **0.142** |  | **Average** |  |  | **0.055** |

Supplementary Table 4 Gene flow calculated by cross-validated SNPs in ipyrad data sets. The parameter -a and -f in BayesAss were set to 0.3 and 0.1 in ipyrad data set, and were set to 0.4 and 0.4 in Stacks data set.

|  | Recipient of migration | | |  |  |  |  |  |  |  |
| --- | --- | --- | --- | --- | --- | --- | --- | --- | --- | --- |
| Source of | Sampling locations |  | MCP | BSH | CEN | JYZ | LB | | QSH | QCP |
| migration | MCP |  |  |  |  |  | LBE | LBI |  |  |
|  |  |  | 0.8984 | 0.0168 | 0.0144 | 0.0139 | 0.0211 | 0.0221 | 0.0138 | 0.0272 |
|  | BSH |  | 0.0144 | 0.8835 | 0.0146 | 0.0136 | 0.0208 | 0.0224 | 0.0139 | 0.0276 |
|  | CEN |  | 0.0145 | 0.0164 | 0.8987 | 0.0138 | 0.0619 | 0.0219 | 0.0136 | 0.0279 |
|  | JYZ |  | 0.0145 | 0.0168 | 0.0148 | 0.9032 | 0.1044 | 0.0445 | 0.014 | 0.0287 |
|  | LB | LBE | 0.0147 | 0.0165 | 0.0144 | 0.014 | 0.7290 | 0.0224 | 0.0137 | 0.0277 |
|  |  | LBI | 0.0143 | 0.0164 | 0.0142 | 0.0138 | 0.0210 | 0.8217 | 0.0139 | 0.0279 |
|  | QSH |  | 0.0146 | 0.0168 | 0.0146 | 0.0138 | 0.0206 | 0.0227 | 0.9029 | 0.0274 |
|  | QCP |  | 0.0146 | 0.0167 | 0.0144 | 0.0138 | 0.0211 | 0.0223 | 0.0142 | 0.8055 |

Supplementary Table 5 The analysis of molecular variance (AMOVA) for two SNP data sets (Stacks data set, ipyrad data set) among four genetic groups (HM, LBI, QSH, QCP).

| Source of variantion | Sum of squares Stack/ipyrad | | Variance components Stack/ipyrad | Percentage variation Stack/ipyrad | *F*_ST_ Stack/ipyrad |
| --- | --- | --- | --- | --- | --- |
| Among populations | | 6226.630/423.404 | 66.59770/4.52329 | 27.08/26.82 | 0.27084/0.26819 |
| Within populations | | 32632.419/2246.322 | 179.29900/12.34243 | 72.92/73.18 |  |
| Total | | 38859.048/2669.726 | 245.89671/16.86572 |  |  |

Supplementary Table 6 Genetic differentiation coefficient (*F*_ST_) between each sampling location and the two genetic groups in LB. The lower triangle is the *F*_ST_ calculated by cross-validated SNPs from the Stacks data set, and the upper triangle is *F*_ST_ calculated by cross-validated SNPs from the ipyrad data set.

| Sampling locations |  | MCP | BSH | CEN | JYZ | LB | | QSH | QCP |
| --- | --- | --- | --- | --- | --- | --- | --- | --- | --- |
|  |  |  |  |  |  | LBE | LBI |  |  |
| MCP |  |  | 0.0532 | 0.0431 | 0.0510 | 0.0673 | 0.1068 | 0.0911 | 0.1522 |
| BSH |  | 0.0498 |  | 0.0447 | 0.0512 | 0.0679 | 0.1156 | 0.1029 | 0.1753 |
| CEN |  | 0.0388 | 0.0393 |  | 0.0453 | 0.0602 | 0.0963 | 0.0898 | 0.1588 |
| JYZ |  | 0.0470 | 0.0474 | 0.0404 |  | 0.0639 | 0.1026 | 0.0899 | 0.1569 |
| LB | LBE | 0.0614 | 0.0606 | 0.0524 | 0.0570 |  | 0.1504 | 0.1989 | 0.4380 |
|  | LBI | 0.1026 | 0.1084 | 0.0874 | 0.0952 | 0.1374 |  | 0.1280 | 0.2518 |
| QSH |  | 0.0844 | 0.0944 | 0.0808 | 0.0828 | 0.1825 | 0.1159 |  | 0.2567 |
| QCP |  | 0.1442 | 0.1622 | 0.1441 | 0.1491 | 0.4124 | 0.2331 | 0.2348 |  |

Supplementary Table 7 Genetic statistics calculated by two cross-validated SNPs data sets.

| Stacks |  |  |  |  |  |
| --- | --- | --- | --- | --- | --- |
| Genetic groups | Sampling locations | *H*_o_ | *H*_e_ | π | *F*_IS_ |
|  | MCP | 0.06544 | 0.07308 | 0.07567 | 0.04472 |
|  | BSH | 0.06741 | 0.07469 | 0.07806 | 0.03648 |
|  | CEN | 0.06084 | 0.06898 | 0.07146 | 0.04569 |
|  | JYZ | 0.06069 | 0.06892 | 0.07121 | 0.04766 |
|  | LB(LBE) | 0.06466 | 0.07008 | 0.07519 | 0.02494 |
| HM |  | 0.06328 | 0.07855 | 0.07917 | 0.14428 |
| LBI | LB(LBI) | 0.07140 | 0.04256 | 0.04597 | -0.04697 |
| QSH | QSH | 0.05442 | 0.06455 | 0.06679 | 0.03707 |
| QCP | QCP | 0.04050 | 0.02835 | 0.03240 | -0.01472 |
| ALL |  | 0.06901 | 0.09051 | 0.09112 | 0.20328 |
| ipyrad |  |  |  |  |  |
| Genetic groups | Sampling locations | *H*_o_ | *H*_e_ | π | *F*_IS_ |
|  | MCP | 0.05939 | 0.07289 | 0.07573 | 0.06679 |
|  | BSH | 0.06252 | 0.07485 | 0.07875 | 0.05195 |
|  | CEN | 0.05532 | 0.06815 | 0.07103 | 0.06151 |
|  | JYZ | 0.05477 | 0.06862 | 0.07120 | 0.06896 |
|  | LB(LBE) | 0.06318 | 0.07024 | 0.07622 | 0.03160 |
| HM |  | 0.05802 | 0.07891 | 0.07961 | 0.19098 |
| LBI | LB(LBI) | 0.06621 | 0.04116 | 0.04479 | -0.03890 |
| QSH | QSH | 0.04984 | 0.06291 | 0.06555 | 0.04455 |
| QCP | QCP | 0.03665 | 0.02527 | 0.02986 | -0.01190 |
| ALL |  | 0.06354 | 0.09117 | 0.09185 | 0.25711 |
